# Investigation of physiological and molecular mechanisms conferring diurnal variation in auxinic herbicide efficacy

**DOI:** 10.1371/journal.pone.0238144

**Published:** 2020-08-28

**Authors:** Christopher R. Johnston, Anish Malladi, William K. Vencill, Timothy L. Grey, A. Stanley Culpepper, Gerald Henry, Mark A. Czarnota, Taylor M. Randell

**Affiliations:** 1 Department of Crop & Soil Sciences, University of Georgia, Athens, GA, United States of America; 2 Department of Horticulture, University of Georgia, Athens, GA, United States of America; 3 Department of Crop & Soil Sciences, University of Georgia, Tifton, GA, United States of America; 4 Department of Horticulture, University of Georgia, Griffin, GA, United States of America; University of Minnesota, UNITED STATES

## Abstract

The efficacy of auxinic herbicides, a valuable weed control tool for growers worldwide, has been shown to vary with the time of day in which applications are made. However, little is known about the mechanisms causing this phenomenon. Investigating the differential *in planta* behavior of these herbicides across different times of application may grant an ability to advise which properties of auxinic herbicides are desirable when applications must be made around the clock. Radiolabeled herbicide experiments demonstrated a likely increase in ATP-binding cassette subfamily B (ABCB)-mediated 2,4-D and dicamba transport in Palmer amaranth (*Amaranthus palmeri* S. Watson) at simulated dawn compared to mid-day, as dose response models indicated that many orders of magnitude higher concentrations of N-1-naphthylphthalamic acid (NPA) and verapamil, respectively, are required to inhibit translocation by 50% at simulated sunrise compared to mid-day. Gas chromatographic analysis displayed that ethylene evolution in *A*. *palmeri* was higher when dicamba was applied during mid-day compared to sunrise. Furthermore, it was found that inhibition of translocation via 2,3,5-triiodobenzoic acid (TIBA) resulted in an increased amount of 2,4-D-induced ethylene evolution at sunrise, and the inhibition of dicamba translocation via NPA reversed the difference in ethylene evolution across time of application. Dawn applications of these herbicides were associated with increased expression of a putative 9-cis-epoxycarotenoid dioxygenase biosynthesis gene *NCED1*, while there was a notable lack of trends observed across times of day and across herbicides with *ACS1*, encoding 1-aminocyclopropane-1-carboxylic acid synthase. Overall, this research indicates that translocation is differentially regulated via specific protein-level mechanisms across times of application, and that ethylene release, a chief phytotoxic process involved in the response to auxinic herbicides, is related to translocation. Furthermore, transcriptional regulation of abscisic acid involvement in phytotoxicity and/or translocation are suggested.

## Introduction

Growers are frequently challenged by variation in herbicide efficacy across the time of day when an application is made. Reduced weed control resulting from this variation is not only detrimental from a financial standpoint, but also has serious implications on the evolution of resistance as reduced herbicide efficacy is consistently linked to selection for resistance-conferring traits [1–3]. Furthermore, each sequential herbicide application necessary to make up for reduced control of previous applications provides an additional selection event. Of particular concern in the southeastern United States is resistance in Palmer amaranth (*Amaranthus palmeri* S. Watson), a weed species that produces a large amount of genetic variability in offspring due to massive seed production and obligate outcrossing [4]. This characteristic coupled with a high growth rate, and thus minimized time required for reproduction, allows for accelerated evolution of herbicide resistance in the presence of overreliance on certain herbicide mechanisms of action [5,6]. Consistently, weeds in the *Amaranthus* genus have already evolved resistance to glyphosate, protoporphyrinogen oxidase inhibitors, acetolactate synthetase inhibitors, 4-hydroxyphenylpyruvate dioxygenase inhibitors, auxinic herbicides, very-long-chain fatty-acid inhibitors, and herbicides of the triazine class [7–13]. The resistance of *A*. *palmeri* to glyphosate in particular has become extremely widespread and problematic for growers [1].

Auxinic herbicides were the first selective herbicides discovered, of which widespread use began with 2,4-dichlorophenoxyacetic acid (2,4-D) [14,15]. 3,6-dichloro-2-methoxybenzoic acid (dicamba) has just recently received a magnitude of use not previously observed in the United States due to the advent of dicamba-resistant row crops such as cotton and soybean, as well as new formulations of dicamba aimed at reducing volatility [16–19]. Metabolic resistance to 2,4-D has also been developed in crops utilizing low volatility formulations of the herbicide [16,20,21]. The advances in herbicide-resistant crops thus warrants extensive study into application strategies that maximize their efficacy. Variation in auxinic herbicide efficacy across time of application has been observed, displaying the classical trend of reduced phytotoxicity near dawn and/or dusk that has been reported with other herbicides [16,22,23]. This has been specifically observed in *A*. *palmeri* under controlled laboratory conditions [24]. Coupled with the aforementioned growth and reproductive characteristics in *Amaranthus* spp., it can thus be conceived that it is only a matter of time until widespread selection for auxinic herbicide-resistant alleles are realized in *Amaranthus* spp. should the increasing auxinic herbicide usage not be associated with maximized efficacy. Not surprisingly, metabolic resistance to 2,4-D has already been reported in waterhemp [*Amaranthus tuberculatus* (Moq.) J.D. Sauer] [25]. Proper stewardship of these herbicides is therefore highly warranted to prevent similar situations from occurring where weeds of the *Amaranthus* genus are widespread.

The causal mechanism(s) responsible for the diurnal variation in auxinic herbicide efficacy have yet to be conclusively established. An increase in translocation has been observed with dawn applications of 2,4-D and dicamba, therefore suggesting an inverse relationship between translocation with herbicidal activity [16]. This suggested relationship contrasts the previously established understanding of the relationship between translocation and phytotoxicity, which classically couples reduced translocation with reduced herbicidal activity [16,26–28]. The framework for the translocation of auxinic herbicides is formed by the activity of auxin transport mechanisms, as structural similarities render auxinic herbicides substrates to many of the same proteins involved in both intra- and intercellular endogenous auxin movement. Identified auxin efflux carriers include proteins of the PIN family and ATP-binding cassette subfamily B (ABCB). PIN proteins are responsible for a portion of the overall framework of intercellular auxin movement, and are strategically positioned in a unipolar fashion to maintain one-directional auxin movement from cell to cell [29–33]. The mechanism of PIN function has been established to be controlled by a proton gradient [34]. In contrast with PIN proteins, members of the ABCB family are mechanistically driven by ATP hydrolysis and are generally associated with the plasma membrane in a nonpolar orientation [35–37]. ABCB and PIN proteins are known to act cooperatively in an additive and synergistic manner [38,39].

Several inhibitors of auxin transport are available that inhibit different portions of the auxin transport framework. The compound N-1-naphthylphthalamic acid (NPA) is known to exhibit inhibitory activity on both PIN and ABCB protein function, potentially through competitive binding/inhibition with both proteins, although it has been noted that inhibition is stronger on PIN activity [31,40]. The compound 2,3,5-triiodobenzoic acid (TIBA) has been shown to selectively block the trafficking of PIN proteins to the plasma membrane resulting in a loss of unidirectional auxin flow [27,41]. In contrast, 5-[*N*-(3,4-dimethoxyphenylethyl)methylamino]-2-(3,4-dimethoxyphenyl)-2-isopropylvaleronitrile hydrochloride (verapamil), a calcium-channel blocker, interacts right at the substrate binding site of ABCB proteins resulting in inhibition of ABCB function [42–46]. These inhibitors of auxin transport can be employed at different times of auxinic herbicide application to grant insight into the functional differences in 2,4-D and dicamba transport proteins across times of day.

The degree of translocation is perhaps also associated and/or correlated with the role of ethylene in the phytotoxic response to auxinic herbicides. Ethylene has been known to be a major contributor to this phytotoxicity for decades, and is tightly associated with herbicide-induced epinasty [47–50]. Ethylene is a trigger for many plant processes, including abscission and response to stress [51]. Auxinic herbicides mimic endogenous auxins in plants and are known to upregulate the synthesis of 1-aminocyclopropane-1-carboxylic acid synthase (ACS), which carries out the rate-limiting step in the biosynthesis of ethylene via the production of the intermediate 1-aminocyclopropane-1-carboxylic acid (ACC) [52–56]. Ultimately, ethylene is reported to induce abscisic acid (ABA) accumulation resulting in inhibition of growth [57]. Since *ACS* expression is directly induced by auxins at the target site, it can be hypothesized that any reduced saturation of the target site with auxins (including auxinic herbicides) due to the aforementioned increased translocation at dawn would result in suppressed ethylene production and thus reduced herbicidal activity. Interestingly, phytochrome responses have also been noted to modulate auxin sensitivity, which may also contribute to the time of day effect [58].

While the ABA-induced upregulation of NADPH-oxidases has been reported to stimulate the formation of reactive oxygen species, the classical stomatal closure and photosynthesis inhibition granted by this hormone are believed to be among the chief herbicidal processes resulting from auxinic herbicide application [51,59–63]. As previously mentioned, ethylene itself has been suggested to stimulate the formation of ABA precursors, illustrating interplay between the pathways associated with both hormones [57]. While substantial research has investigated the overall roles of ethylene and abscisic acid in the herbicidal response to auxinic materials, none have examined the degree of related gene expression corresponding to applications at different times of day.

Overall, determining the relationship of gene expression and ethylene evolution with functional differences in auxin transport mechanisms may provide a mechanistic understanding into the time of day phenomenon observed with auxinic herbicides. The objectives of this research were to 1) determine if differential effects of auxin transport inhibitors across times of day are present, 2) establish a relationship between herbicide-induced ethylene production and herbicide translocation via application at different simulated times of day and with translocation-inhibiting compounds, and 3) determine the association of ABA and ethylene biosynthesis genes’ transcript abundance with different application times.

## Materials and methods

### Plant materials

A confirmed glyphosate-resistant *Amaranthus palmeri* (S. Wats.) population collected from Macon Co., GA in 2015 was used for all experiments. Seeds were germinated in potting mix (Fafard 3B Mix, Sun Gro Horticulture, Agawam, MA) in 55 × 28 ×10 cm growth trays. Plants were germinated and established in a growth chamber set to a day/night temperature of 30/20°C with light from 8 am to 12 am at 600 μmol m^-2^ s^-1^ and 50% relative humidity. Plants were selected that reached ~15 cm in height prior to the beginning of experiments to control for developmental stage and growth rate. For translocation and ethylene production experiments, selected plants were then transplanted into 125 ml Nalgene bottles (Thermo Fisher Scientific, Waltham, MA) with deionized water used as growth medium. A 20-20-20 N-P-K fertilizer was added to each bottle at a concentration equivalent to 1/6 the nitrogen content of a full-strength Hoagland solution (210 ppm). For gene expression experiments, selected plants were kept in the same potting mix in which they were germinated. With all experiments, plants ready for treatment were placed under an LED light (Kind LED K5 Series, Kind LED Grow Lights, Santa Rosa, CA) program under laboratory conditions at 21°C, with light spectrum and intensity settings modified to be consistent with those observed in early spring (S1 Fig) as established by the manufacturer. Plants were allowed to acclimate to these conditions for 48 h prior to treatment.

### Application of treatments

#### Translocation experiments

For translocation experiments, the experimental design was a factorial arrangement of the three inhibitors at three concentrations each (1, 10, and 25 μM) and two application times (8 am and 1 pm, representing simulated dawn and mid-day, respectively). Separate experiments were performed for both 2,4-D and dicamba. Four replications were used and experiments were repeated in time to provide two experimental runs total (each run referred to as “experiment 1” and “experiment 2”). Treatments included controls receiving herbicide but no inhibitor, and no herbicide or inhibitor. Plants were treated with inhibitors dissolved in dimethyl sulfoxide (DMSO) via delivery to growth solution prior to herbicide treatments. Final concentration of DMSO in growth solution following these inhibitor treatments did not exceed 0.1% v/v and inhibitors were added 8 h before herbicide application to ensure uptake, both consistent with previous research on 2,4-D translocation inhibition which was verified prior to these experiments [27]. Regardless of inhibitor treatment, plants were sprayed with the diglycolamine salt of dicamba at 0.19 kg a.e. ha^-1^ (Xtendimax with VaporGrip Technology, Monsanto Co.) while the amine salt of 2,4-D was applied at 0.23 kg a.e. ha^-1^ (2,4-D Amine 4-D, Dow Chemical) using a pressurized backpack sprayer calibrated to deliver 280 L ha^-1^. Rates were determined in preliminary experiments to ensure translocation of herbicide without excess phytotoxicity to prevent premature tissue death. Plants had one leaf covered with plastic film prior to the backpack application, which was removed following the drying of spray droplets. This leaf was then treated with 1.79 kBq of the choline salt of ^14^C-2,4-D (ring-labeled, specific activity 7.449 MBq mg^-1^) for 2,4-D experiments and 1.05 kBq of the diglycolamine salt of ^14^C-dicamba (ring-labeled, specific activity 1.658 MBq mg^-1^) for dicamba experiments. These labeled herbicides were used to trace movement in response to different inhibitors and times of day. The broadcast application of non-labeled herbicide prior to ^14^C-herbicide application was conducted to simulate the application of a typical spray volume of the total active ingredient in the field.

#### Ethylene experiments

For ethylene experiments, inhibitors were only added prior to simulated dawn applications at only the maximum 25 μM concentration in accordance with a completely randomized design. Inhibitors were used at dawn only in order to determine if reducing translocation at a time it is normally found to be higher reversed the time of application effect. Separate experiments were performed for both 2,4-D and dicamba, and four replications were used with experiments repeated in time to provide two experimental runs total. Treatments thus included 2,4-D or dicamba application at simulated dawn and mid-day, and dawn applications with one of three inhibitors as previously mentioned. Plants were treated with NPA, TIBA, or verapamil dissolved in DMSO via delivery to growth solution 8 h prior to herbicide treatments. Final concentration of DMSO in growth solution following these inhibitor treatments did not exceed 0.1% v/v. Plants were sprayed with the amine salt of 2,4-D at 0.70 kg a.e. ha^-1^ or the diglycolamine salt of dicamba at 0.38 kg a.e. ha^-1^ using a pressurized backpack sprayer calibrated to deliver 280 L ha^-1^. Rates were determined in preliminary experiments to ensure detectable ethylene release without premature plant death during the gas sampling period (see below).

#### Gene expression experiments

No inhibitors were used for gene expression experiments, and thus the only two treatments were dawn and mid-day herbicide applications. Four replications were used and experiments were repeated in time to provide two experimental runs total. Dicamba and 2,4-D treatments were both included in each experiment. 2,4-D and dicamba were applied at 0.21 kg a.e. ha^-1^ and 0.17 kg a.e. ha^-1^, respectively, with a pipettor in 7–10 μl drops to simulate the 280 L ha^-1^ coverage, with drops evenly divided across the number of plant leaves. A pipettor was used to ensure the same amount of herbicide was intercepted per plant, controlling for variation in leaf area present during broadcast applications.

### Determination of ^14^C-labeled herbicide translocation

Plants were removed from bottles 48 h after ^14^C-labeled herbicide applications in accordance with previous research and immediately sectioned into ^14^C-treated leaves, nontreated leaves, stems, and roots [16]. Treated leaves were rinsed twice with 1 ml of a 10% aqueous methanol solution to remove any unabsorbed ^14^C-labeled herbicide. Following the rinse, all plant sections were dried for 72 h at 80°C. Treated leaves and stems were then run through a biological oxidizer (OX500, RJ Harvey Instrument Corp., Hillsdale, NJ) and the radioactivity associated with the evolved ^14^CO_2_ from each plant fraction was trapped in a ^14^C-cocktail. This radioactivity was measured using liquid scintillation spectrometry (Tri-Carb 2910 TR, PerkinElmer Inc., Waltham, MA). It was found that 105 and 97% of applied radioactivity was recovered from treated leaves and stems of 2,4-D and dicamba experiments, respectively, therefore roots and nontreated leaves were not included in the analysis. Absorption of ^14^C-labeled herbicide was quantified as the percentage of applied radioactivity recovered from leaf wash or inside plant parts. Translocation was quantified as the percentage of absorbed radioactivity found in the stem. Absorption data were subjected to GLM in JMP (JMP Pro 13, SAS Institute, Cary, NC) and translocation data were analyzed using nonlinear regression in R (R v. 3.4.4, R Foundation for Statistical Computing, Vienna, Austria) via the ‘drc’ and ‘qpcR’ packages [64,65]. Model selection was carried out based on AICc scores (S Info.). In the event of convergence failures of models, experimental runs were separated and models were fitted to each experimental run individually. Models and associated statistical parameters were plotted using Sigmaplot (Sigmaplot 11, Systat Software, San Jose, CA).

### Determination of herbicide-induced ethylene production

Treated plants were placed in 1,893 ml jars and sealed with metal lids 3 h prior to sampling. Each metal lid contained a rubber septum for sampling and an AA battery pack connected to a 2.5 cm-diameter fan, both sealed to the lid with silicone (S2 Fig). Fans were used to ensure adequate air mixing in the jar headspace. Rubber septa were pierced with a 1 ml insulin syringe and 1 ml of headspace gas was collected per measurement. Headspace samples were then injected into a gas chromatograph coupled with a flame ionization detector (GC-FID) (Shimadzu GC-17A, Shimadzu Corp., Kyoto, Japan) using helium as carrier gas with a 10 ml min^-1^ flow rate and a micro-packed HayeSep N column (Restek Corp., Bellefonte, PA). The temperature program was 60°C for 4 min followed by a 15°C min^-1^ increase to a hold at 150°C. For 2,4-D experiments, samples were taken at 14, 20, 28 and 36 hours after treatment (HAT) and for dicamba experiments samples were taken at 14, 20, and 28 HAT. Time points were established based on a preliminary experiment that identified the time of initiation of an herbicide-induced ethylene pulse (S3 Fig). A standard curve was used to estimate all ethylene concentrations which were then converted to μl kg FW^-1^ h^-1^ (S4 Fig). ANCOVA was applied to data using JMP with HAT serving as the covariate and the log of ethylene production (transformed so linear relationships could be established) as the response variable. The special ANCOVA assumptions of linear relationship of dependent variable and covariate with each treatment, along with a lack of covariate by treatment interactions (see below), were thus met in addition to normal assumptions of ANOVA. Mean ethylene production was separated across treatments using pairwise t-tests and were presented using SigmaPlot.

### RNA extraction, purification, and cDNA synthesis

Plants were harvested 3 h after herbicide treatment, consistent with the time reported to maximize upregulation of 9-cis-epoxycarotenoid dioxygenase (*NCED*) genes involved in ABA biosynthesis [66]. Approximately 0.4 g of pulverized frozen leaf tissue was added to 3 ml of a 2% cetyl trimethylammonium bromide (CTAB) extraction buffer (S Info.). The mixture was homogenized and incubated at 65°C for 10 min followed by another homogenization and incubation for 5 min at room temperature (23°C). Phase separation was carried out with a 24:1 chloroform:isoamyl alcohol solution and was centrifuged at 5,000 g for 5 min prior to precipitation overnight in 0.25 volumes of LiCl at 4°C. A cold 70% ethanol solution in DEPC-treated water was used to rinse prior to incubation in 1× SSTE buffer and cold absolute ethanol for 2 h. RNA was further rinsed with 70% cold ethanol and air dried on ice prior to re-dissolution in DEPC-treated water. Quantification of RNA concentration was carried out using a NanoDrop (NanoDrop 8000, Thermo Fisher Scientific, Waltham, MA), and quality confirmation was performed via gel electrophoresis by observing intactness of 18S and 28S bands. RNA was subjected to a DNase cycle (Promega) to remove genomic DNA contamination, and reverse transcription was subsequently performed using oligo dT (Promega) and reverse transcriptase (ImPromII, Promega). DNase and reverse transcriptase treatments were performed according to manufacturer instructions.

### Identification and sequencing of NCED1

The *NCED3* sequence of thale cress [*Arabidopsis thaliana* (L.) Heynh.] was obtained from NCBI (National Center for Biotechnology Information, U.S. National Library of Medicine, Bethesda, MD) and used as a query against the prince’s-feather (*Amaranthus hypochondriacus* L.) genome using Phytozome (Phytozome 12, The Regents of the University of California, Oakland, CA). The resulting *A*. *hypochondriacus* scaffolds with the lowest E value between the two species were aligned using Clustal Omega (Multiple Sequence Alignment Tool, European Molecular Biology Laboratory—European Bioinformatics Institute, Cambridgeshire, UK). Primers were designed at areas of significant scaffold matching at the 5’ and 3’ end (S Info.) of this sequence. Primers were tested in a PCR with 2 μl of cDNA (50 ng uL^-1^) from 2,4-D-treated plants. The primer test PCR was a hot-start run with 40 cycles consisting of denaturation at 95°C for 30 s, annealing at 60°C for 30 s, and elongation at 72°C for 1 min.

The primer pair, *NCED3F* 5’-GGTCATCATTTCTTTGACGGTGA-3’ and *NCED3R* 5’-AATCCAGACACCTTTGGCCA-3’, yielded a ~1,000 bp product (S4 Fig). This product was extracted from the gel and sequenced at the Georgia Genomics Facility (Georgia Genomics and Bioinformatics Core, Athens, GA) using Sanger capillary sequencing and the PCR amplification primers. The resulting sequence was then analyzed using Sequence Manipulation Suite Translate Tool [67] (S Info.). BLAST analysis of the predicted protein sequence indicated 86% amino acid identity with *NCED1* from *Chenopodium quinoa* Willd., a member of the Amaranthaceae family which includes *A*. *palmeri* (query cover = 99%, E-value = 0.0). The nucleotide sequence for this *NCED1* in *A*. *palmeri* was submitted to NCBI (Accession No.: MT108369). Gene-specific qPCR primers were then designed using sequence information.

### qPCR and expression analysis

A 2X SYBR Green master mix (PowerUp SYBR Green Master Mix, Applied Biosystems, Foster City, CA) with a ROX reference was used to perform qPCR analyses. The reaction was carried out in a qPCR system (Mx3005P, Stratagene, San Diego, CA). A dissociation profile was examined following amplification to ensure specificity of primer pairs. The 18S ribosomal RNA gene (*18SRibo*) of *A*. *palmeri* (Accession No.: MG685258.1) was used as a reference gene, utilizing the primers *Forward* 5'-AGTGGATGCACCCAGTATT-3' and *Reverse* 5'-TCGATGGTTCACGGGATT-3'. The primers *Forward* 5’-GATCGTCGTAATCGGATCTTG-3’ and *Reverse* 5’-TCTCTCCGGGTTGAAACT-3’ were selected for *NCED1* amplification. *ACS1* qPCR primers used were *Forward* 5’-AAGCTGGATGGTTTAGAGTATG-3’ and *Reverse* 5’-GATGCCAACATTTCCTCTTTG-3’, which were designed based on the available *A*. *Palmeri* transcriptome (Accession No.:SRX7295363) (S Info.) [68]. The amplified qPCR product sizes ranged from 120–200 bp.

Amplification efficiency was determined using LinRegPCR [69], with the overall efficiency averaged within genes and experiments used for calculation of respective relative quantity of transcript (RQ) values. Values for RQ, normalized relative quantity of transcript (NRQ), log2-transformed NRQ data (Cq`) and standard errors of relative transcript expression (RE) were calculated according to [70].

## Results

### Herbicide translocation

**Fig 1:**
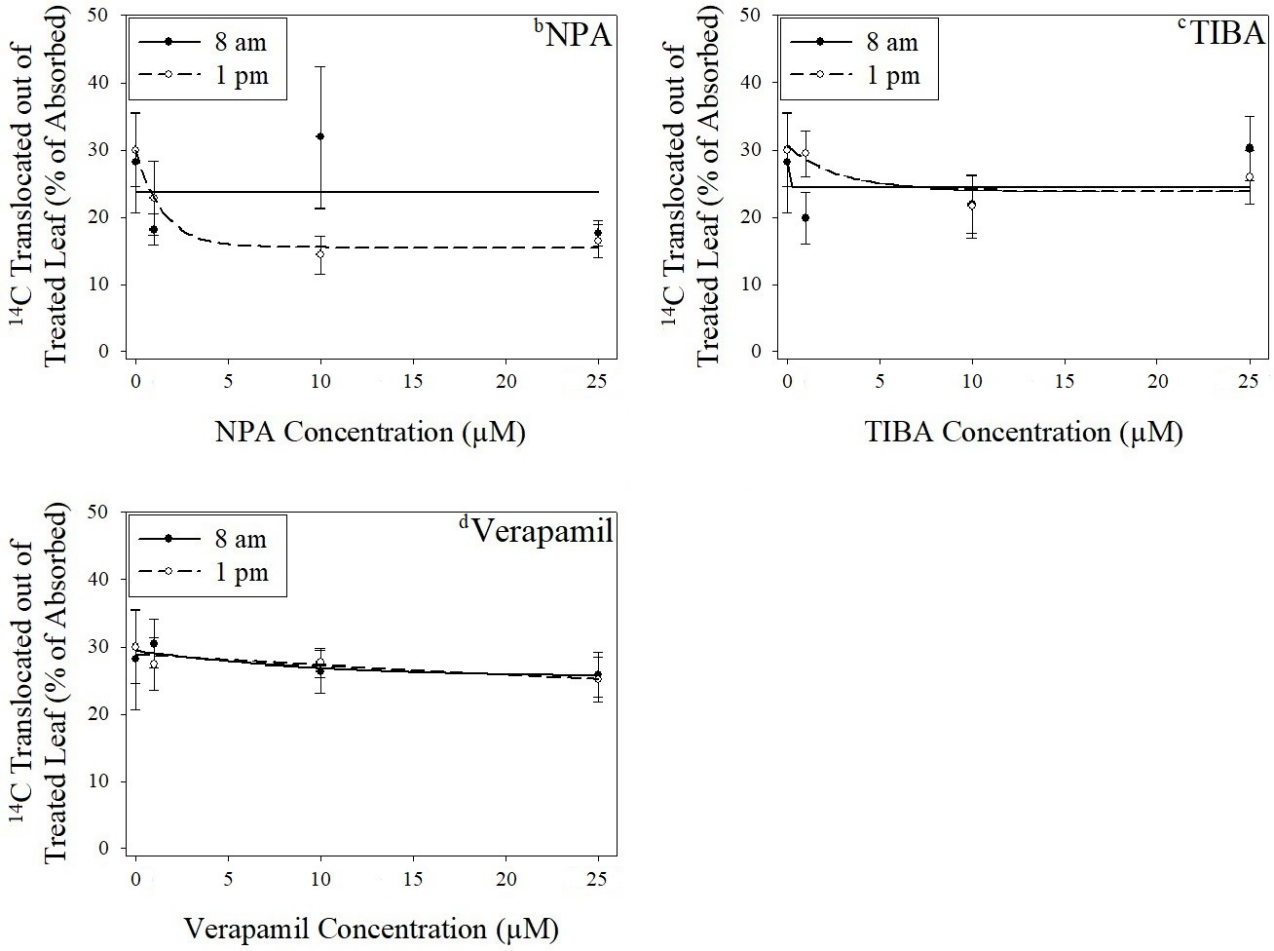
Effect of increasing translocation inhibitor concentrations on translocation of ^14^C-2,4-D from treated leaves. ^a^Vertical bars represent standard error of the mean. ^b^NPA = N-1-naphthylphthalamic acid. ^c^TIBA = 2,3,5-triiodobenzoic acid. ^d^Verapamil = 5-[*N*-(3,4-dimethoxyphenylethyl)methylamino]-2-(3,4-dimethoxyphenyl)-2-isopropylvaleronitrile hydrochloride. ^e^NPA with 8 am ^14^C-2,4-D application: Standard error of regression (SER) = 18.09; NPA with 1 pm ^14^C-2,4-D application: SER = 11.58; TIBA with 8 am ^14^C-2,4-D application: SER = 14.44; TIBA with 1 pm ^14^C-2,4-D application: SER = 11.81; Verapamil with 8 am ^14^C-2,4-D application: SER = 12.28; Verapamil with 1 pm ^14^C-2,4-D application: SER = 10.42.

For 2,4-D and dicamba experiments, an average of 66% and 53% of applied herbicide was absorbed by the plant, respectively (S1 Table). Experimental run by rate or application time interactions were not detected for any inhibitor in 2,4-D experiments; therefore data are combined within each inhibitor. For all inhibitors in 2,4-D experiments, the exponential decay function was used. Steepness of decay, given by parameter *e*, was significantly different across application times with NPA (p < 0.0001); therefore the null hypothesis that models were the same across application times was rejected (S2 Table and Fig 1). The TI_50_ (i.e. ED_50_, dose resulting in a 50% decrease in translocation) for NPA was significantly lower with 2,4-D application at 1 pm at 1.02 μM, compared with 2.27 × 10^10^ μM for 2,4-D application at 8 am. Relative potency of 2,4-D application at 1 pm with respect to 8 am was thus < 0.0001, indicating a greater amount of NPA was necessary for reduction of translocation at 8 am. No significant differences were detected for parameters across application times with TIBA or verapamil.

**Fig 2:**
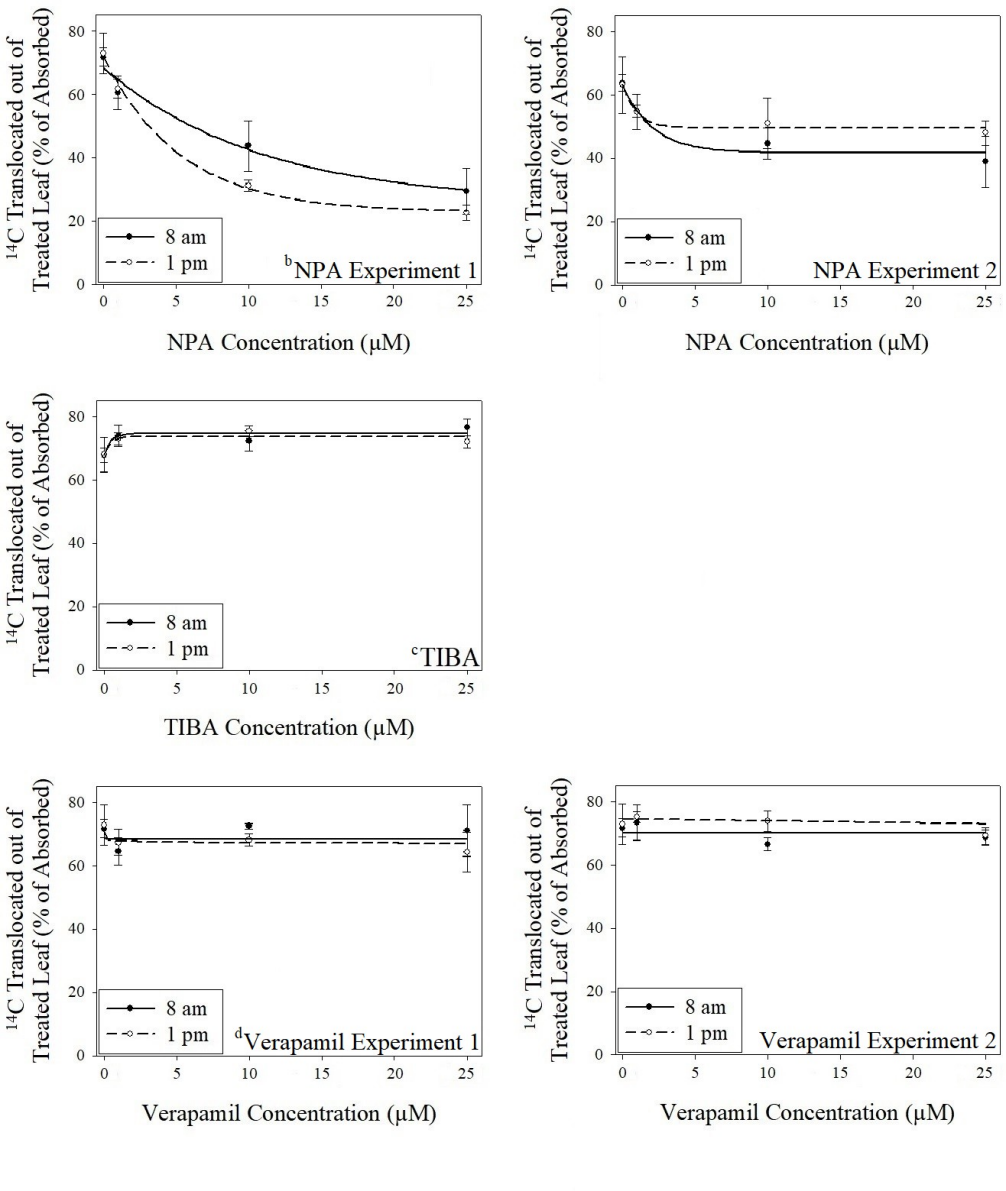
Effect of increasing translocation inhibitor concentrations on translocation of ^14^C-dicamba from treated leaves. ^a^Vertical bars represent standard error of the mean. ^b^NPA = N-1-naphthylphthalamic acid. ^c^TIBA = 2,3,5-triiodobenzoic acid. ^d^Verapamil = 5-[*N*-(3,4-dimethoxyphenylethyl)methylamino]-2-(3,4-dimethoxyphenyl)-2-isopropylvaleronitrile hydrochloride. ^e^NPA with 8 am ^14^C-dicamba application, experiment 1: Standard error of regression (SER) = 12.27; NPA with 1 pm ^14^C-dicamba application, experiment 1: SER = 7.43; NPA with 8 am ^14^C-dicamba application, experiment 2: SER = 9.91; NPA with 1 pm ^14^C-dicamba application, experiment 2: SER = 13.30; TIBA with 8 am ^14^C-dicamba application: SER = 8.20; TIBA with 1 pm ^14^C-dicamba application: SER = 8.81; Verapamil with 8 am ^14^C-dicamba application, experiment 1: SER = 13.32; Verapamil with 1 pm ^14^C-dicamba application, experiment 1: SER = 10.14; Verapamil with 8 am ^14^C-dicamba application, experiment 2: SER = 7.40; Verapamil with 1 pm ^14^C-dicamba application, experiment 2: SER = 7.54.

An experimental run by rate interaction was detected for NPA in dicamba experiments (p = 0.0124); therefore comparisons of NPA models were carried out separately for each experimental run. No experimental run by treatment interactions were detected for TIBA or verapamil. Therefore, models for TIBA are combined over experimental runs; however, a model convergence failure for combined verapamil data resulted in separate model fitting for each experimental run. No significant parameter differences were detected across application times for NPA or TIBA; however, for experiment 1 with verapamil, the *I*_*50*_ (inflection point) was significantly different across application times (p < 0.0001), and for experiment 2 with verapamil, both *T*_*min*_ (lower limit of translocation at full inhibitor dose) and *e* were significantly different (p < 0.0001) (S3 Table and Fig 2). As a result, the null hypothesis was rejected for the effect of verapamil across application times in both experiments. In experiment 1, the TI_50_ for verapamil with an 8 am application of dicamba was 3.32 × 10^15^ μM compared to 0.33 μM for the 1 pm application, and in experiment 2, the TI_50_ with the 8 am application was 2.75 × 10^9^ μM compared to 827 μM with the 1 pm application. Relative potency for 1 pm applications with respect to 8 am applications was < 0.0001 for both experiments. Significant differences in the *I*_*50*_ and *e* parameters, and subsequently a higher TI_50_ for verapamil with dicamba application at dawn in experiments 1 and 2, respectively, clearly indicate that a higher concentration of verapamil is necessary for dicamba transport to be inhibited at this time. It is important to note that for both dicamba experiments, the parameters associated with the verapamil analyses fall well outside of the concentration range used for study. As a result, the estimation of such parameters is likely of low resolution.

### Ethylene production

**Fig 3:**
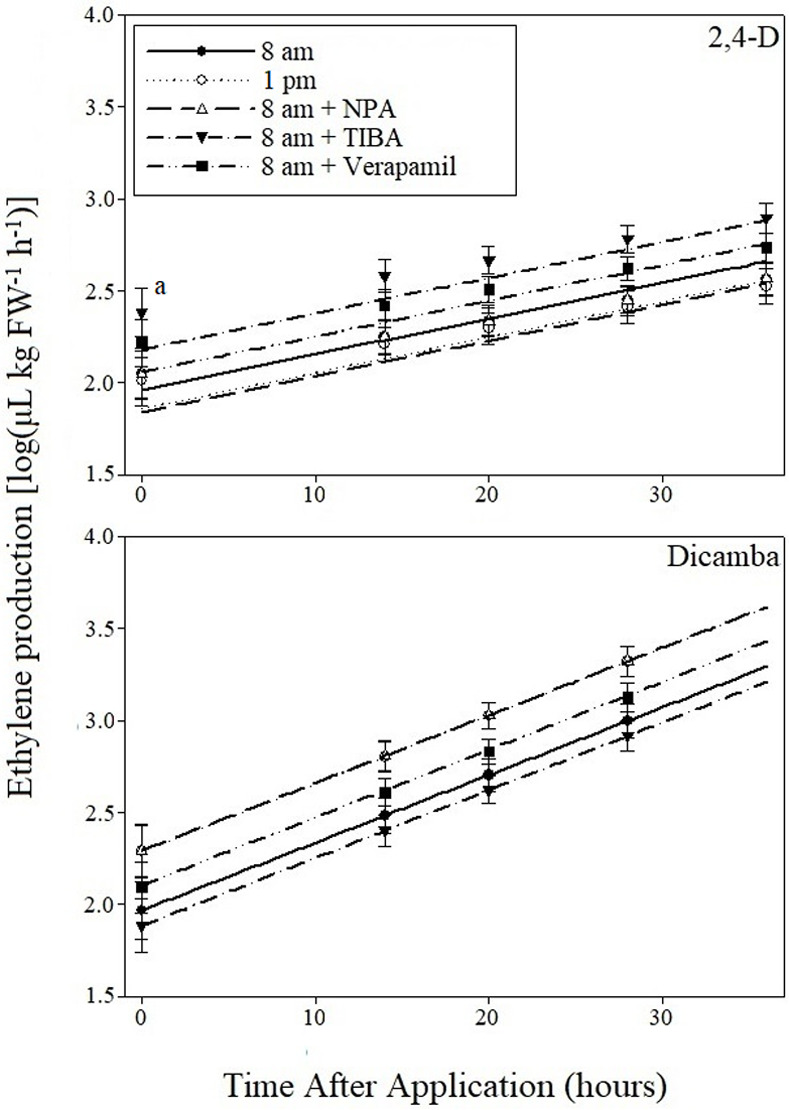
Prediction equations of ethylene production resulting from 2,4-D and dicamba applications at a rate of 0.70 and 0.38 kg a.e. ha^-1^, respectively, applied at two timings and with three translocation inhibitors. ^a^Vertical bars represent standard error of the mean. Bars at zero hours after application represent standard error of the predicted intercept.

Covariate by treatment interactions were not detected indicating lack of significant differences in slope across treatments for 2,4-D experiments (Table 1 and Fig 3). A highly significant effect of the covariate was detected along with a significant effect of treatment. No significant differences between the 1 pm application and 8 am application with no pre-treatment were detected. The 8 am application pre-treated with TIBA resulted in the highest ethylene production at 2.69 μL kg FW^-1^ h^-1^, which was significantly greater than all treatments except for the 8 am application pre-treated with verapamil. The 8 am treatment pre-treated with NPA resulted in the least ethylene production at 2.35 μL kg FW^-1^ h^-1^, significantly less than the 8 am application pre-treated with TIBA or verapamil. The lack of a significant difference in ethylene production between the 8 am application with no pre-treatment and the 1 pm application suggest a potential lack of differential phytotoxicity across application times with 2,4-D.

**Table 1:** Analysis of covariance results from 2,4-D applications at a rate of 0.70 kg a.e. ha^-1^, applied at two timings and with three translocation inhibitors.

Treatment	Mean (SE) log (μL kg FW^-1^ h^-1^)		Equation	Slope^a^ (SE)
1:00 PM	2.36 (0.15)	CD^b^	y = 1.86 + 0.019x	0.0194 (0.0044)
8:00 AM	2.37 (0.14)	BCD	y = 1.96 + 0.019x	__
8 am + TIBA	2.69 (0.14)	A	y = 2.18 + 0.019x	__
8 am + NPA	2.35 (0.14)	D	y = 1.84 + 0.019x	__
8 am + Verapamil	2.56 (0.14)	ABC	y = 2.06 + 0.019x	__
HAT^c^	<0.0001			
Treatment	0.0019			
HAT*Treatment	0.3806			

^a^Covariate by treatment interactions were not detected, therefore one slope is reported for all treatments.

^b^Means followed by different letters are significantly (p ≤ 0.05) different. Means separations are based on pairwise t-tests.

^c^HAT = hours after treatment.

The treatment by covariate interaction was statistically insignificant for dicamba experiments, yielding one slope for all treatments (Table 2 and Fig 3). The greatest ethylene production was noted with the 1 pm application and 8 am application pretreated with NPA, both with a mean of 3.07 μL kg FW^-1^ h^-1^. Significant differences were detected between the 1 pm treatment and 8 am treatment with no inhibitor. The 8 am application pre-treated with TIBA resulted in the least ethylene production at 2.66 μL kg FW^-1^ h^-1^.

**Table 2:** Analysis of covariance results from dicamba applications at a rate of 0.38 kg a.e. ha^-1^, applied at two timings and with three translocation inhibitors.

Treatment	Mean (SE) log (μL kg FW^-1^ h^-1^)		Equation	Slope^a^ (SE)
1:00 PM	3.07 (0.07)	A^b^	y = 2.29 + 0.037x	0.0368 (0.0059)
8:00 AM	2.75 (0.09)	B	y = 1.97 + 0.037x	__
8 am + TIBA	2.66 (0.07)	B	y = 1.88 + 0.037x	__
8 am + NPA	3.07 (0.07)	A	y = 2.29 + 0.037x	__
8 am + Verapamil	2.87 (0.07)	AB	y = 2.10 + 0.037x	__
HAT^c^	<0.0001			
Treatment	0.0004			
HAT*Treatment	0.6661			

^a^Covariate by treatment interactions were not detected, therefore one slope is reported for all treatments.

^b^Means followed by different letters are significantly (p ≤ 0.05) different. Means separations are based on pairwise t-tests.

^c^HAT = hours after treatment

### Gene expression

**Fig 4:**
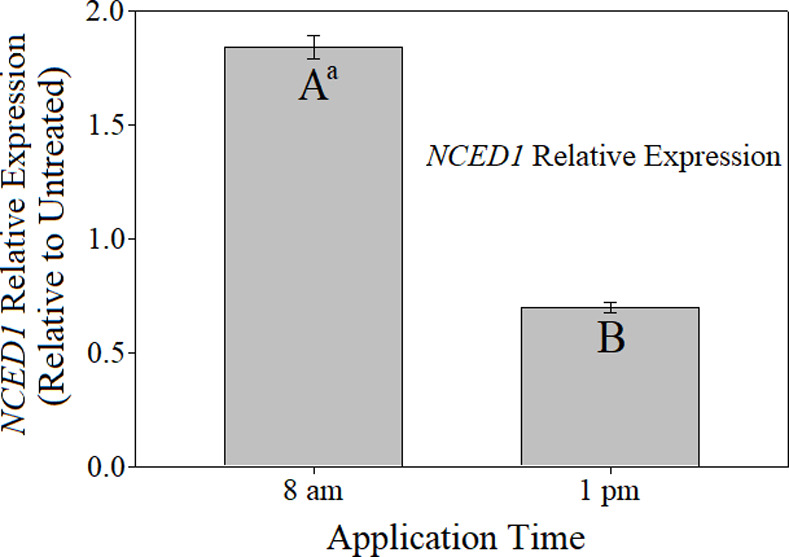
Relative expression of *NCED1* resulting from morning and mid-day herbicide applications relative to nontreated control. ^a^Means followed by different letters differ significantly according to student’s t-test at α = 0.05.

**Fig 5:**
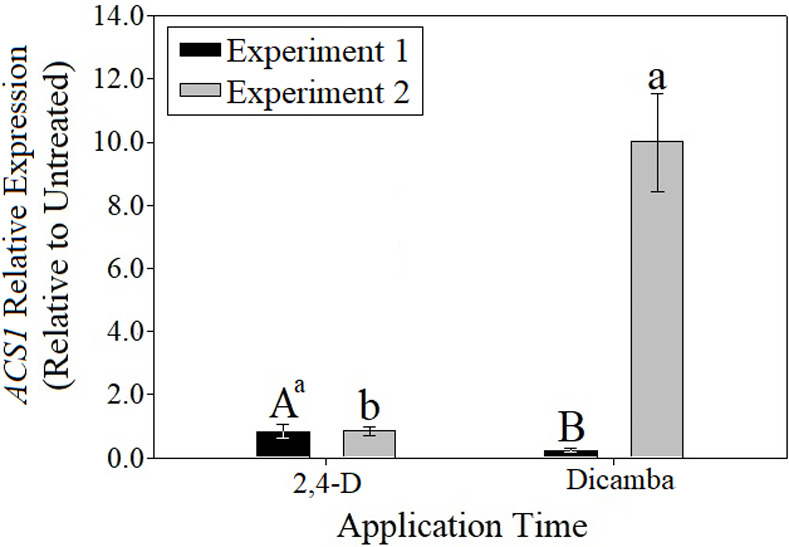
Relative expression of *ACS1* resulting from 2,4-D and dicamba applications relative to nontreated control. ^a^Means followed by different letters differ significantly according to student’s t-test at α = 0.05. Uppercase letters correspond to comparisons within experiment one, whereas lowercase letters correspond to comparisons within experiment two.

No significant herbicide effects were detected for *NCED1* data (p = 0.3696), however significant differences were detected between the relative expression associated with the two times of application (p = 0.0494) (S4 Table and Fig 4). Relative expression of *NCED1* was approximately three-fold higher when herbicide applications were made at 8 am compared to 1 pm. These results demonstrate a clear association of application time and *NCED1* activity, indicating some degree of diurnal control on herbicide-inducible ABA biosynthesis at the transcriptional level. Experimental run by herbicide interactions were detected for *ACS1* expression; therefore, experiments were analyzed and are presented separately. In experiment one, relative expression of *ACS1* was significantly higher with 2,4-D compared to dicamba application by 3.5 fold (S5 Table and Fig 5). In contrast, with experiment two, relative expression from dicamba application was significantly higher than 2,4-D. Expression of *ACS1* with dicamba application in experiment two was nearly 45 times higher than that observed with dicamba in experiment one.

## Discussion

**Fig 6:**
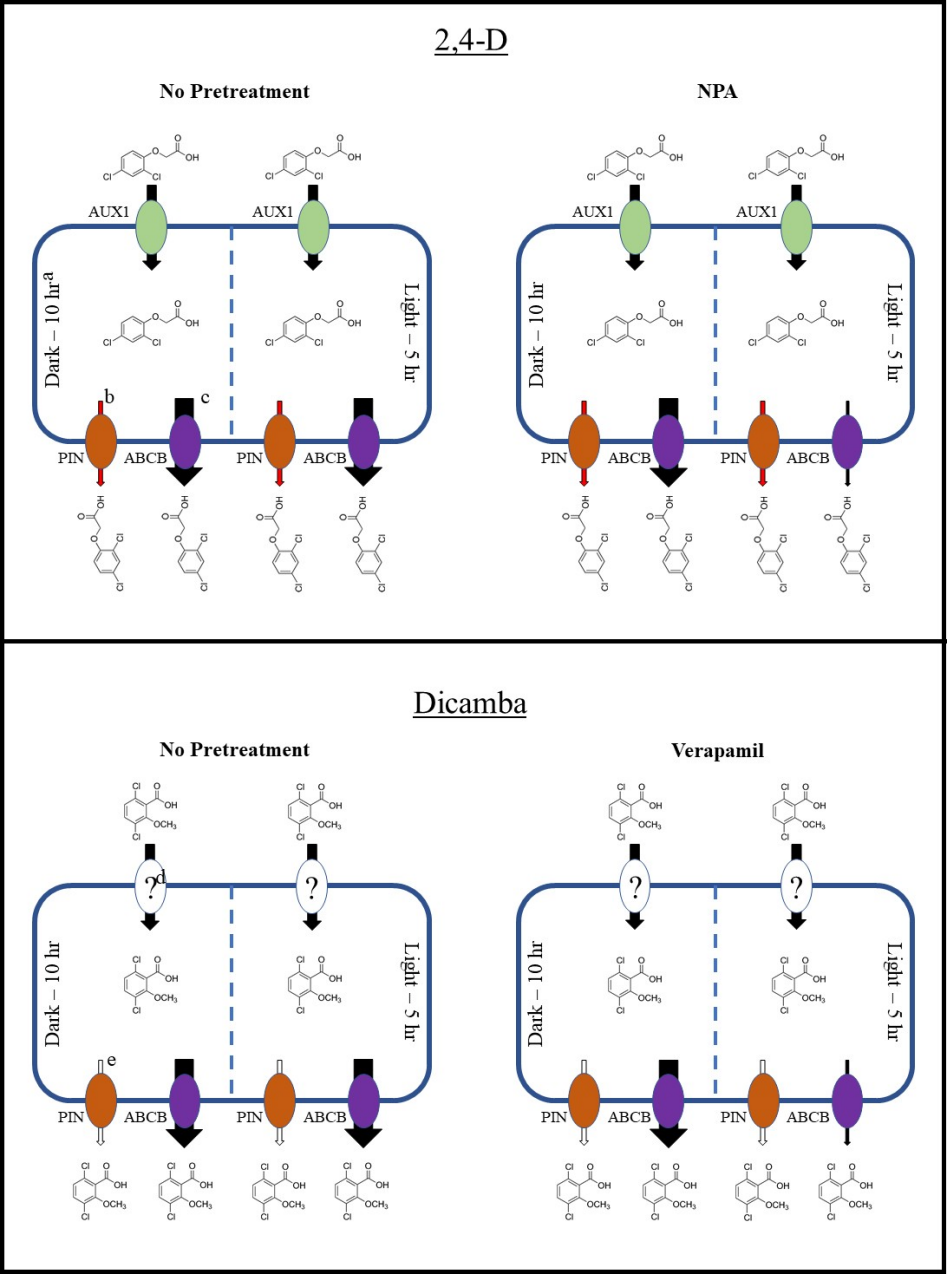
Diagrams illustrating differences in 2,4-D and dicamba efflux from plant cells across application times with no pre-treatment and NPA and verapamil pretreatment, respectively. ^a^Dark– 10 h indicates the 8 am, simulated dawn application, as applications were made after 10 h of darkness. Light– 5 h indicates the 1 pm, simulated mid-day application, as applications were made after 5 h of light. ^b^Red arrows indicate probable 2,4-D efflux via PIN transport proteins. ^c^Size of black arrows indicate magnitude of efflux via ABCB proteins. ^d^Question mark indicates unknown influx carrier, if any, of dicamba. ^e^White arrows indicate unknown extent, if any, of dicamba efflux via PIN proteins.

The same concentration of NPA had a much larger effect on 2,4-D translocation at 1 pm compared to 8 am, which is indicative of a greater abundance of respective transport proteins expressed at dawn and is consistent with herbicide translocation trends in previous research [16]. *PIN3* expression is known to be upregulated by active phytochrome B (mid-day) via two genes encoding transcription factors that are members of a helix-loop-helix protein family, phytochrome-interacting factor 4 (*PIF4*) and *PIF5* [71–75]. Transcript accumulation of *PIF4* specifically is generally only present during the day, with peak transcript levels occurring at mid-day [73,76]. This fact coupled with a lack of differential activity of the PIN-specific inhibitor TIBA across application times suggests that PIN activity is not the limiting 2,4-D transport process inhibited by NPA in this research. This is, however, operating under the assumption that transcription and protein levels/activity are directly and quickly correlated with transcription. Whether or not this is the case in *A*. *palmeri* requires further investigation, as PIN protein activity is regulated at many post-transcriptional levels [32]. It has been reported that 2,4-D transport is not dependent on PIN proteins, and the findings in this research are consistent with these results [77]. It can therefore be suggested that the critical NPA-sensitive 2,4-D transport function being inhibited in this research is the ABCB transporter complex (Fig 6). Long-distance transport of 2,4-D in wild radish (*Raphanus raphanistrum* L.) and *Arabidopsis* has been linked to ABCB transporters [27,78].

Part of the differences in activity of NPA and verapamil in these findings may be due to the mechanism of action of both inhibitors. NPA has been demonstrated to bind directly with low affinity to not only ABCB1 and ABCB19, but also with high affinity to a regulatory protein TWD1 which is responsible for localization of several ABCB proteins to the plasma membrane, required for auxin transport functionality [35,79–82]. In contrast, verapamil is a calcium channel blocker but also shows direct binding at the substrate site of ABCB proteins [42,44]. Consistent with this research, ABCB transport function has been shown to be less sensitive to verapamil than NPA [35,83,84]. As such, this may be the reason behind reduced activity of verapamil on inhibition of 2,4-D transport compared to NPA, as well as a lack of significant differences in activity across 2,4-D application time seen with verapamil. Higher concentrations are thus potentially necessary to produce this effect with verapamil. This is inconsistent with previous research in which verapamil at a 10 μM concentration for root uptake demonstrated a significant degree of inhibition of 2,4-D transport in *R*. *raphanistrum* [27]. Indeed, as the translocation proteins mentioned are members of multigene families, affinity of the aforementioned inhibitors for each member may differ, thus resulting in differing concentrations/compounds being necessary for the desired activity in *A*. *palmeri* in particular.

Little diurnal variation in *TWD1*, *ABCB1*, *ABCB4*, and *ABCB19* transcript abundance is observed across long-day conditions similar to those used in this research, suggesting some other mechanism is providing increased sensitivity to NPA of ABCB-mediated 2,4-D transport at dawn [85]. The previously reported increase in 2,4-D translocation at dawn is consistent with the findings of decreased sensitivity to NPA at this time in this research [16]. Additionally, since TIBA did result in reductions of 2,4-D transport, some PIN-mediated 2,4-D transport is likely occurring, despite previous literature showing that 2,4-D movement is not completely reliant on PIN proteins [77].

Previous work has illustrated that flavonol competition with dicamba for ABCB transport resulted in reduced dicamba translocation in kochia [*Bassia scoparia* (L.) A.J. Scott] [86]. As such, it is well-substantiated, along with the results of this research, that dicamba is a substrate for ABCB transporters. Furthermore, the lack of differences in dicamba translocation with increasing TIBA concentrations suggests either a negligible amount or lack of PIN protein-mediated dicamba translocation. Interestingly, NPA, which as previously stated has activity on both PIN and ABCB transporters, failed to yield a significant difference in dicamba translocation across application times in both experiments. As previously mentioned, since *ABCB* and *TWD1* genes display little variation in expression across time of day, a post-translational mechanism conferring differential ABCB activity may be at work. The interpretations of verapamil activity on herbicide translocation needs to be taken very reluctantly due to the fact that effective activity was not observed within the range of concentrations tested experimentally, therefore resulting in a low confidence estimation of associated parameters.

**Fig 7:**
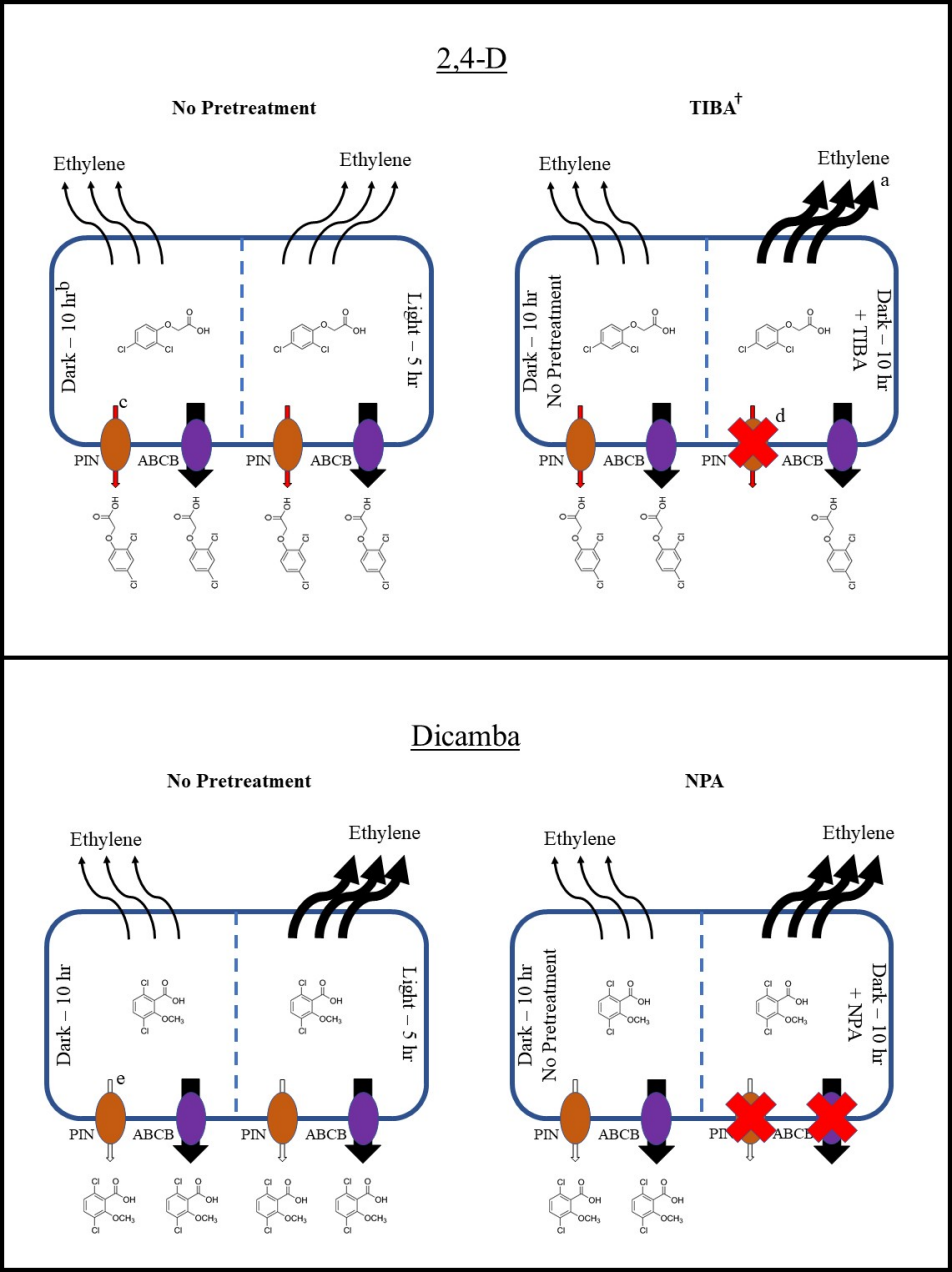
Diagrams illustrating differences in 2,4-D and dicamba-induced ethylene production from plant cells across application times, and across 8 am applications with no pretreatment and TIBA and NPA pretreatment, respectively. ^a^Size of black arrows indicate magnitude of ethylene production. ^b^Dark– 10 h indicates the 8 am, simulated dawn application, as applications were made after 10 h of darkness. Light– 5 h indicates the 1 pm, simulated mid-day application, as applications were made after 5 h of light. ^c^Red arrows indicate probable 2,4-D efflux via PIN transport proteins. ^d^Red “X” indicates likely total inhibition of efflux via respective transport protein. ^e^White arrows indicate unknown extent, if any, of dicamba efflux via PIN proteins.

A potential lack of ethylene-related differential phytotoxicity across application times with 2,4-D is suggested in this research (Fig 7). Should a true difference in phytotoxicity occur across application times for another reason, it may be due to differential abscisic acid (ABA) accumulation or radical oxygen species (ROS) evolution. The evolution of ROS in particular would have major implications here, as it is one of the main phytotoxic processes resulting from herbicide application [50]. Further investigation is necessary to determine if any differential 2,4-D perception at the target site is present across application times. Nevertheless, the findings of this research provide an important insight into the relationship of translocation with phytotoxicity. A lack of the ability to translocate 2,4-D may result in an improved opportunity/duration for the herbicide to ultimately activate the auxin response factors associated with the signal cascade resulting from auxinic herbicide application [87,88]. This may provide the mechanistic basis for the increased dicamba-induced phytotoxicity observed with certain weed species when the auxin transport inhibitor diflufenzopyr is added [89].

The findings of this research strongly assert that ethylene production is at least one of the defining factors in the time of day effect with dicamba. It is also highly noteworthy that NPA completely reversed the differences in ethylene production observed across time of application. This directly suggests that the reduced translocation previously reported with mid-day dicamba applications is one of the major factors in differential phytotoxicity [16], as shutting down of cellular dicamba efflux via NPA resulted in a statistically significant increase in ethylene production.

Inhibition of auxin efflux has been shown in previous research to enhance the defoliation induced by exogenously applied ethylene, presumably in accordance with a hormone balance model [90,91]. Specifically, the inhibitory effect of ethylene itself on auxin supply to cells is coupled with an ethylene-induced increase in hydrolases that aid in the cell separation responsible for abscission [92,93]. The pre-treatment of plants with auxin has been reported to reverse the defoliation activity of ethylene [90]. Since a lack of auxinic herbicide translocation would thus limit the balance of auxin and ethylene at the whole-plant level, phytotoxic responses in addition to defoliation may indeed be enhanced via this hormone balance model.

Intuitively, it would be expected that due to the negative effects on plant growth imparted by increased ABA production, higher *NCED1* expression would be detected with mid-day applications when these herbicides are more active [60,94]. Several reasons may be behind the contradictory observations found in this research. ABA promotes stomatal closure, resulting in a reduction in carbon fixation and a potential overall reduction in photosynthesis, which may in turn negatively affect rates of general biochemical activity [95–97]. This reduction may reduce the perception of auxinic herbicides when applied at dawn, as well as decrease the degree of signal transduction caused by this perception. Consistently, previous research has illustrated that auxin activity, specifically by inhibiting lateral root formation, is antagonized by ABA [98]. This may be only one of potentially a whole suite of auxin-inducible process affected by ABA. Furthermore, ABA has been shown to increase auxin conjugation, thus reducing overall free concentrations of auxin in the cell via regulation of storage and transport [99]. In contrast, when plants are attacked by auxinic herbicides at a time with lower ABA activity (i.e. mid-day), a full degree of perception and therefore signal transduction may be allowed to occur. However, this hypothesis relies on the assumption that increased *NCED1* expression does indeed cause a direct increase in ABA, disregarding the potential activity of post-translational regulation mechanisms and metabolic manipulation of ABA concentrations in plant cells. Further research is necessary to confirm this phenomenon. Regardless, even if plant biochemical activity is reduced with dawn applications, this does not appear to affect the ability of the plant to move auxinic herbicides [16].

The lack of consistent trends in *ACS1* expression across experiments convolutes the ability to draw any direct conclusions concerning the role of ethylene biosynthesis in auxinic herbicide efficacy. Since no significant time of application effects were detected, there may be another mechanism (in addition to there still being a possibility of differential *ACS1* expression not detected by this research) that causes the reported increase in ethylene production with dicamba applications at mid-day compared to dawn. Additionally, another member(s) of the *ACS* gene family not analyzed here may play a major role in this difference.

## Conclusions

It appears that reductions in auxinic herbicide activity at dawn coincides with an increase in *NCED1* expression and thus potentially increased ABA concentrations in the plant cell. For this reason, the increase in *NCED1* activity not only at dawn, but also potentially under other conditions imparting plant stress (due to increased ABA concentrations), may result in a reduction of herbicide efficacy for growers. As such, it may be recommended that auxinic herbicides be applied not only to plants between an hour after dawn and an hour before dusk, but also to *A*. *palmeri* plants under a negligible amount of stress.

The diurnal variation in activity of ABCB-mediated 2,4-D and dicamba translocation appears to be at least partially responsible for any differential translocation across time of application. Furthermore, this research identifies an auxin translocation-dependent mechanism(s) that controls ethylene production, a major process in the phytotoxic effect of auxinic herbicides. While there is some evidence that ABCB protein abundance and/or functionality is differentially augmented across time of day, it remains to be elucidated whether or not this occurs at the transcriptional level with *A*. *palmeri* (in contrast with *Arabidopsis*) or at the post-transcriptional and -translational level. Identification of this mechanism may provide valuable information as to whether certain auxinic chemistries display a lesser degree of diurnal variation in translocation and/or activity than others. This knowledge would provide applicable implications for further development of auxinic herbicide-resistant crops, and ultimately further potential strategies for growers working with large acreage or in adverse environments that rely on herbicide applications to be made at different times of day.

## Supporting information

S1 Fig LED light program used for *A*. *palmeri* plants in experiments. ^a^PPFD = Photosynthetic photon flux density. ^b^Red light range = 635–685 nm; Far-red light range = 710–760 nm. ^c^Black arrows represent both simulated dawn (8 am) and mid-day (1 pm) application times of 2,4-D and dicamba.
(TIF)

S2 Fig Jars used for ethylene collected from *A*. *palmeri* plants treated with 2,4-D and dicamba. (TIF)

S3 Fig Ethylene production resulting from 0.46 kg a.e. ha^-1^ 2,4-D application at 1 pm, determined from preliminary experiment to evaluate initial peak rate of herbicide-induced ethylene production. ^a^Vertical bars represent standard error of the mean. ^b^Cubic polynomial function fit to data, following the formula: *y = -3*.*15 + 0*.*36x + -0*.*0061x*^*2*^ *+ 0*.*000042x*^*3*^. SE = 2.81, r^2^ = 0.37.
(TIF)

S4 Fig Initial amplification of potential *NCED* gene product in *A*. *palmeri* using gel electrophoresis. 1.2% agarose in TBE buffer. ^a^Potential *NCED* product amplified using *NCED3* sequence from *A*. *thaliana*. 1× strength PCR reaction mixture, ~1,000 bp product. Primer sequences: F 5’-GGTCATCATTTCTTTGACGGTGA-3’ and R 5’-AATCCAGACACCTTTGGCCA-3’. ^b^*β-tubulin* used for reference gene. Primer sequences: F 5'-ATGTGGGATGCCAAGAACATGATGTG-3' and R 5'-TCCACTCCACAAAGTAGGAAGAGTTCT-3'.
(TIF)

S5 Fig *NCED1* gene product used for DNA extraction, isolated using gel electrophoresis. 1.2% agarose in TBE buffer. ^a^*NCED1* product amplified using a 2× strength PCR reaction mixture, ~1,000 bp. Primer sequences: F 5’-GGTCATCATTTCTTTGACGGTGA-3’ and R 5’-AATCCAGACACCTTTGGCCA-3’. ^b^DNA excised from gel isolated using gel extraction kit (E.Z.N.A. Gel Extraction Kit, Omega Bio-Tek, Norcross, GA) and sequenced via Sanger capillary sequencing.
(TIF)

S1 Table Nonlinear regression results and dose response analysis of ^14^C-2,4-D experiments. (PDF)

S2 Table Nonlinear regression results and dose response analysis of ^14^C-dicamba experiments. (PDF)

S3 Table Absorption of ^14^C-herbicides in *A*. *palmeri* translocation experiments. (PDF)

S4 Table Relative expression values of *NCED1* resulting from morning and mid-day herbicide applications, relative to untreated control. (PDF)

S5 Table Relative expression values of *ACS1* resulting from morning and mid-day herbicide applications, relative to untreated control. (PDF)

S1 File Additional supporting information. (PDF)

S1 Raw images (PDF)

S1 Data (ZIP)
