# Supplementary figures and images for: Investigation of physiological and molecular mechanisms conferring diurnal variation in auxinic herbicide efficacy
Source: PLoS One. 2020 Aug 28;15(8):e0238144. doi: 10.1371/journal.pone.0238144 (PMC7454982; doi:10.1371/journal.pone.0238144)

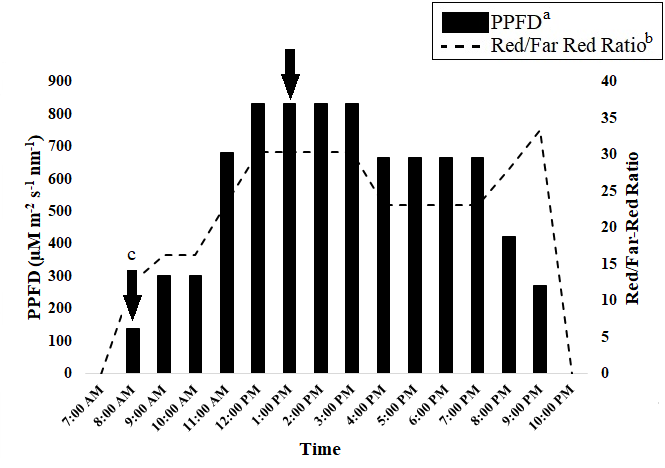

Supplement: S1 Fig — aPPFD = Photosynthetic photon flux density. bRed light range = 635–685 nm; Far-red light range = 710–760 nm. cBlack arrows represent both simulated dawn (8 am) and mid-day (1 pm) application times of 2,4-D and dicamba. (TIF) [file pone.0238144.s001.tif]

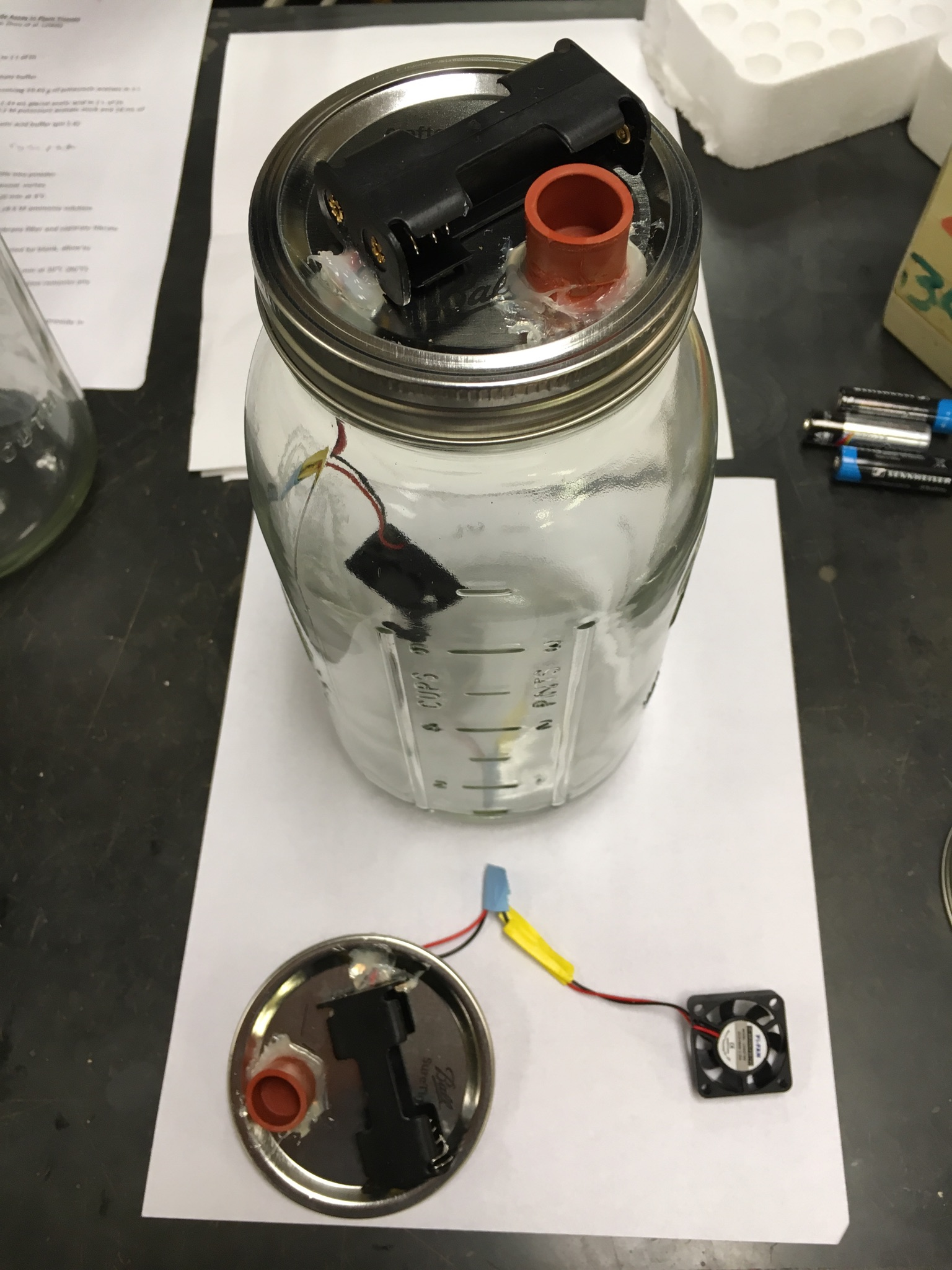

Supplement: S2 Fig — (TIF) [file pone.0238144.s002.tif]

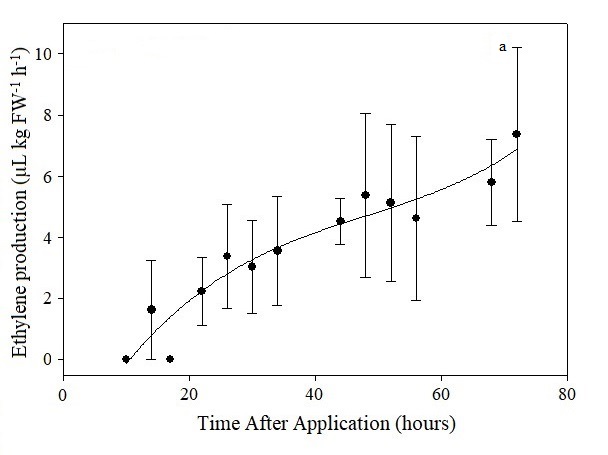

Supplement: S3 Fig — aVertical bars represent standard error of the mean. bCubic polynomial function fit to data, following the formula: y = -3.15 + 0.36x + -0.0061x2 + 0.000042x3. SE = 2.81, r2 = 0.37. (TIF) [file pone.0238144.s003.tif]

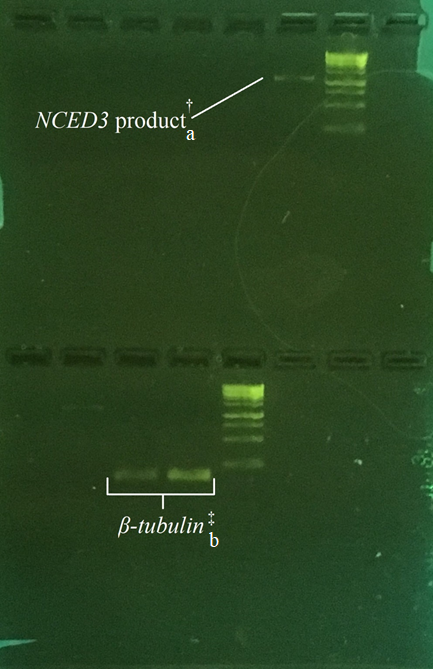

Supplement: S4 Fig — 1.2% agarose in TBE buffer. aPotential NCED product amplified using NCED3 sequence from A. thaliana. 1× strength PCR reaction mixture, ~1,000 bp product. Primer sequences: F 5’-GGTCATCATTTCTTTGACGGTGA-3’ and R 5’-AATCCAGACACCTTTGGCCA-3’. bβ-tubulin used for reference gene. Primer sequences: F 5'-ATGTGGGATGCCAAGAACATGATGTG-3' and R 5'-TCCACTCCACAAAGTAGGAAGAGTTCT-3'. (TIF) [file pone.0238144.s004.tif]

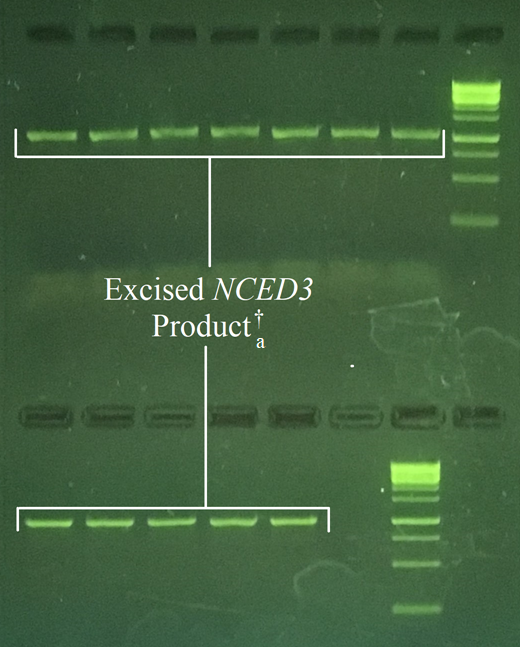

Supplement: S5 Fig — 1.2% agarose in TBE buffer. aNCED1 product amplified using a 2× strength PCR reaction mixture, ~1,000 bp. Primer sequences: F 5’-GGTCATCATTTCTTTGACGGTGA-3’ and R 5’-AATCCAGACACCTTTGGCCA-3’. bDNA excised from gel isolated using gel extraction kit (E.Z.N.A. Gel Extraction Kit, Omega Bio-Tek, Norcross, GA) and sequenced via Sanger capillary sequencing. (TIF) [file pone.0238144.s005.tif]
