# Supplementary material for: Investigation of physiological and molecular mechanisms conferring diurnal variation in auxinic herbicide efficacy
Source: PLoS One. 2020 Aug 28;15(8):e0238144. doi: 10.1371/journal.pone.0238144 (PMC7454982; doi:10.1371/journal.pone.0238144)
Supplement: S1 Table — (PDF) [file pone.0238144.s006.pdf]

Experiment	Inhibitor	Rate	Absorption
			%
1	[a]NPA	1 µM	68 [b](3.33)
		10 µM	77 (5.24)
		25 µM	71 (2.69)
	[c]Verapamil	1 µM	71 (4.44)
		10 µM	70 (3.07)
		25 µM	65 (1.47)
	[d]TIBA	1 µM	74 (1.97)
		10 µM	71 (2.26)
		25 µM	70 (2.06)
	None		74 (1.77)
2			
	NPA	1 µM	60 (4.38)
		10 µM	57 (5.23)
		25 µM	75 (2.07)
	Verapamil	1 µM	64 (2.36)
		10 µM	68 (3.89)
		25 µM	59 (2.98)
	TIBA	1 µM	52 (4.74)
		10 µM	67 (5.21)
		25 µM	56 (5.76)
	None		53 (3.95)

Table 1. Absorption of ^{14}C-2,4-D in response to increasing rates of translocation inhibitors, 2018.

[a]NPA = N-1-naphthylphthalamic acid.

[b]Parentheses represent standard error of the mean.

[c]Verapamil = 5-[*N*-(3,4-dimethoxyphenylethyl)methylamino]-2-(3,4-dimethoxyphenyl)-2isopropylvaleronitrile hydrochloride.

[d]TIBA = 2,3,5-triiodobenzoic acid.

Inhibitor	Rate	Absorption
		%
[a]NPA	1 μM	56 [b](3.71)
	10 μM	56 (3.49)
	25 μM	60 (3.03)
[c]Verapamil	1 μM	47 (3.29)
	10 μM	53 (4.39)
	25 μM	52 (3.09)
[d]TIBA	1 μM	52 (3.58)
	10 μM	57 (4.46)
	25 μM	52 (3.01)
None		43 (4.51)

Table 2. Absorption of ^{14}C-dicamba in response to increasing rates of translocation inhibitors, 2018.

[a]NPA = N-1-naphthylphthalamic acid.

[b]Parentheses represent standard error of the mean.

[c]Verapamil = 5-[*N*-(3,4-dimethoxyphenylethyl)methylamino]-2-(3,4-dimethoxyphenyl)-2isopropylvaleronitrile hydrochloride.

[d]TIBA = 2,3,5-triiodobenzoic acid.
