# Supplementary material for: Investigation of physiological and molecular mechanisms conferring diurnal variation in auxinic herbicide efficacy
Source: PLoS One. 2020 Aug 28;15(8):e0238144. doi: 10.1371/journal.pone.0238144 (PMC7454982; doi:10.1371/journal.pone.0238144)
Supplement: S2 Table — (PDF) [file pone.0238144.s007.pdf]

Inhibitor	Equation	Parameter	[1]p
[2]NPA	$y = T_{min} + (T_0 – T_{min})(exp(-x/e))$	[3]T_{min}	0.3505
		[4]T_0	0.2704
		[5]e	<0.0001
[6]TIBA	$y = T_{min} + (T_0 – T_{min})(exp(-x/e))$	T_{min}	0.9244
		T_0	0.7023
		e	0.6548
[7]Verapamil	$y = T_{min} + (T_0 – T_{min})(exp(-x/e))$	T_{min}	0.97
		T_0	0.8964
		e	0.9698

Table 1. Equations and results of z-tests for comparing parameters between 8 am and 1 pm applications of ^{14}C-2,4-D on Palmer amaranth using different translocation inhibitors, 2018.

[1]p = p-value corresponding to z-test comparing respective parameters between 8 am and 1 pm application. Considered statistically significant at $p \leq 0.05$.

[2]NPA = N-1-naphthylphthalamic acid.

[3]T_{min} = lower limit of translocation according to regression equation.

[4]T_0 = translocation at $x = 0$.

[5]e = steepness of decay.

[6]TIBA = 2,3,5-triiodobenzoic acid.

[7]Verapamil = 5-[*N*-(3,4-dimethoxyphenylethyl)methylamino]-2-(3,4-dimethoxyphenyl)-2-isopropylvaleronitrile hydrochloride.

Inhibitor	Application Time	Parameter	Estimate
[1]NPA	8:00 AM	[2]T_{min}	5.46
		[3]T_0	23.75
		[4]e	3.27E+10
	1:00 PM	T_{min}	15.45
		T_0	30.03
		e	1.48
[5]TIBA	8:00 AM	T_{min}	24.31
		T_0	28.13
		e	0.091
	1:00 PM	T_{min}	23.86
		T_0	30.63
		e	2.81
[6]Verapamil	8:00 AM	T_{min}	25.40
		T_0	29.44
		e	9.70
	1:00 PM	T_{min}	15.54
		T_0	28.86
		e	77.37

Table 2. Parameters associated with exponential decay functions used for regression of ^{14}C-2,4-D translocation in Palmer amaranth at two different application times and with different translocation inhibitors, 2018.

[1]NPA = N-1-naphthylphthalamic acid.

[2]T_{min} = lower limit of translocation according to regression equation.

[3]T_0 = translocation at $x = 0$.

[4]e = steepness of decay.

[5]TIBA = 2,3,5-triiodobenzoic acid.

[6]Verapamil = 5-[*N*-(3,4-dimethoxyphenylethyl)methylamino]-2-(3,4-dimethoxyphenyl)-2-isopropylvaleronitrile hydrochloride.

Inhibitor	Application Time	$^{a}ED_{50}$ (μM)	[b]Relative Potency (1pm/8am)
[c]NPA	8:00 AM	>	
	1:00 PM	1.02	4.52E-11
[4]TIBA	8:00 AM	[5]NS	
	1:00 PM	NS	NS
[6]Verapamil	8:00 AM	NS	
	1:00 PM	NS	NS

Table 3. Dose response analysis comparing effect of increasing translocation inhibitor concentrations between two different application times of ^{14}C-2,4-D in Palmer amaranth, 2018.

$^{a}ED_{50}$ = Effective dose for concentration of inhibitor required to achieve 50% inhibition of translocation.

[b]Relative Potency = Relative index used for comparing ED_{50} across application times, corresponding to quotient of ED_{50} for 1 pm application divided by ED_{50} for 8 am application.

[c]NPA = N-1-naphthylphthalamic acid.

[4]TIBA = 2,3,5-triiodobenzoic acid.

[5]NS = not significant. Dose response analysis was not carried out for inhibitors that failed to yield a significant ($p \leq 0.05$) difference in the z-test between application times with any parameter. Corresponds to a failure to reject the null hypothesis that curve functions were significantly different between application times.

[6]Verapamil = 5-[*N*-(3,4-dimethoxyphenylethyl)methylamino]-2-(3,4-dimethoxyphenyl)-2-isopropylvaleronitrile hydrochloride.
