# Supplementary material for: Investigation of physiological and molecular mechanisms conferring diurnal variation in auxinic herbicide efficacy
Source: PLoS One. 2020 Aug 28;15(8):e0238144. doi: 10.1371/journal.pone.0238144 (PMC7454982; doi:10.1371/journal.pone.0238144)
Supplement: S3 Table — (PDF) [file pone.0238144.s008.pdf]

Inhibitor	Experiment	Equation	Parameter	[1]p
[2]NPA	1	$y = T_{min} + (T_0 - T_{min})(exp(-x/e))$	[3]T_{min}	0.8394
			[4]T_0	0.5747
			[5]e	0.5249
	2	$y = T_{min} + (T_0 - T_{min})(exp(-x/e))$	T_{min}	0.2071
			T_0	0.9586
			e	0.7448
[6]TIBA	Combined	$y = T_0 + (T_{max} - T_0)(1 - exp(-x/e))$	T_0	0.9431
			[7]T_{max}	0.768
			e	0.9785
[8]Verapamil	1	$y = T_{min} + [(T_0 - T_{min})/ (1 + exp(b(log(x) - log(I_{50}))))]$	[9]b	0.5627
			T_{min}	0.3592
			T_0	0.4403
			[10]I_{50}	<0.0001
	2	$y = T_{min} + (T_0 - T_{min})(exp(-x/e))$	T_{min}	<0.0001
			T_0	0.1025
			e	<0.0001

Table 1. Equations and results of z-tests for comparing parameters between 8 am and 1 pm applications of ^{14}C-dicamba on Palmer amaranth using different translocation inhibitors, 2018.

[1]p = p-value corresponding to z-test comparing respective parameters between 8 am and 1 pm application. Considered statistically significant at $p \leq 0.05$.

[2]NPA = N-1-naphthylphthalamic acid.

[3]T_{min} = lower limit of translocation according to regression equation.

[4]T_0 = translocation at $x = 0$.

[5]e = steepness of decay for exponential decay function, and steepness of increase for asymptotic regression function.

[6]TIBA = 2,3,5-triiodobenzoic acid.

[7]T_{max} = Upper limit of translocation.

[8]Verapamil = 5-[*N*-(3,4-dimethoxyphenylethyl)methylamino]-2-(3,4-dimethoxyphenyl)-2isopropylvaleronitrile hydrochloride.

[9]*b* = slope.

[10]I_{50} = inflection point, or dose giving 50% reduction in translocation.

Inhibitor	Experiment	Application Time	Parameter	Estimate
[1]NPA	1	8:00 AM	[2]T_{min}	25.41
			[3]T_0	68.35
			[4]e	10.87
		1:00 PM	T_{min}	22.62
			T_0	71.75
			e	5.34
	2	8:00 AM	T_{min}	41.66
			T_0	63.61
			e	2.10
		1:00 PM	T_{min}	49.53
			T_0	63.16
			e	1.02
[5]TIBA	Combined	8:00 AM	T_0	67.75
			[6]T_{max}	74.62
			e	0.42
		1:00 PM	T_0	68.06
			T_{max}	73.71
			e	0.46
[7]Verapamil	1	8:00 AM	[8]b	0.0076
			T_{min}	47.42
			T_0	86.93
			[9]I_{50}	3.32E+15
		1:00 PM	b	0.13
			T_{min}	59.78
			T_0	76.50
			I_{50}	0.33

2	8:00 AM	T_{min}	56.74
		T_0	70.086
		e	3.96E9
	1:00 PM	T_{min}	-0.015
		T_0	74.52
		e	1193.10

Table 2. Parameters associated with exponential decay, asymptotic regression, or log-logistic functions used for regression of ^{14}C-dicamba translocation in Palmer amaranth at two different application times and with different translocation inhibitors, 2018.

[1]NPA = N-1-naphthylphthalamic acid.

[2]T_{min} = lower limit of translocation according to regression equation.

[3]T_0 = translocation at $x = 0$.

[4]e = steepness of decay for exponential decay function, and steepness of increase for asymptotic regression function.

[5]TIBA = 2,3,5-triiodobenzoic acid.

[6]T_{max} = Upper limit of translocation.

[7]Verapamil = 5-[*N*-(3,4-dimethoxyphenylethyl)methylamino]-2-(3,4-dimethoxyphenyl)-2isopropylvaleronitrile hydrochloride.

[8]b = slope.

[9]I_{50} = inflection point, or dose giving 50% reduction in translocation.

Inhibitor	Experiment	Application Time	[a]ED_{50} (μM)	[b]Relative Potency (1pm/8am)
[c]NPA	1	8:00 AM	[d]NS	
		1:00 PM	NS	NS
	2	8:00 AM	NS	
		1:00 PM	NS	NS
[e]TIBA	Combined	8:00 AM	NS	
		1:00 PM	NS	NS
[f]Verapamil	1	8:00 AM	>	
		1:00 PM	0.33	9.82E-17
	2	8:00 AM	>	
		1:00 PM	827	3.01E-07

Table 3. Dose response analysis comparing effect of increasing translocation inhibitor concentrations between two different application times of ^{14}C-dicamba in Palmer amaranth, 2018.

[a]ED_{50} = Effective dose for concentration of inhibitor required to achieve 50% inhibition of translocation.

[b]Relative Potency = Relative index used for comparing ED_{50} across application times, corresponding to quotient of ED_{50} for 1 pm application divided by ED_{50} for 8 am application.

[c]NPA = N-1-naphthylphthalamic acid.

[d]NS = not significant. Dose response analysis was not carried out for inhibitors that failed to yield a significant ($p \leq 0.05$) difference in the z-test between application times with any parameter. Corresponds to a failure to reject the null hypothesis that curve functions were significantly different between application times. [e]TIBA = 2,3,5-triiodobenzoic acid.

[f]Verapamil = 5-[*N*-(3,4-dimethoxyphenylethyl)methylamino]-2-(3,4-dimethoxyphenyl)-2isopropylvaleronitrile hydrochloride.
