# Supplementary material for: Investigation of physiological and molecular mechanisms conferring diurnal variation in auxinic herbicide efficacy
Source: PLoS One. 2020 Aug 28;15(8):e0238144. doi: 10.1371/journal.pone.0238144 (PMC7454982; doi:10.1371/journal.pone.0238144)
Supplement: S4 Table — (PDF) [file pone.0238144.s009.pdf]

Time of Application	NRQ[a] (SE[b])	Relative Expression (SE)	
8:00 AM	0.104 (0.0278)	1.842 (0.0512)	A[c]
1:00 PM	0.0394 (0.0179)	0.699 (0.0242)	B
Untreated	0.0563 (0.0234)		
	Study*Herbicide	0.23	
	Study*TOA[d]	0.56	
	Herbicide	0.37	
	TOA	0.049	
	Herbicide*TOA	0.19	

Table 1. Expression of *NCED1* resulting from morning and mid-day herbicide applications relative to untreated control, 2018.

[a]NRQ = normalized relative quantity of transcript. Normalized by dividing relative quantity of *NCED1* transcript by relative quantity of *18SRibo*, the reference gene.

[b]SE = standard error of the mean. Standard error for relative expression means calculated as described by the equation:

$$SE\left(\frac{NRQ}{NRQunt}\right) = \left[\frac{NRQ^\wedge 2}{NRQunt^\wedge 2}\left(\frac{SE(NRQ)^\wedge 2}{NRQ^\wedge 2} + \frac{SE(NRQunt)^\wedge 2}{NRQunt^\wedge 2}\right)\right]^{1/2}$$

where *SE* is the standard error of corresponding terms, *NRQ* is the mean normalized relative quantity for each respective treatment, and *NRQunt* is the mean normalized relative quantity for the untreated control.

[c]Means followed by different letters differ significantly according to student’s t-test at $\alpha = 0.05$.

[d]TOA = time of application.
