# Supplementary material for: Investigation of physiological and molecular mechanisms conferring diurnal variation in auxinic herbicide efficacy
Source: PLoS One. 2020 Aug 28;15(8):e0238144. doi: 10.1371/journal.pone.0238144 (PMC7454982; doi:10.1371/journal.pone.0238144)
Supplement: S5 Table — (PDF) [file pone.0238144.s010.pdf]

Experiment	Herbicide	NRQ[a] (SE[b])	Relative Expression (SE)	
1	2,4-D	0.492 (0.159)	0.827 (0.249)	A[c]
	Dicamba	0.134 (0.0739)	0.225 (0.0903)	B
	Untreated	0.595 (0.231)		
		Herbicide	0.029	
		TOA[d]	0.051	
		Herbicide*TOA	0.24	
2	2,4-D	0.176 (0.0490)	0.852 (0.123)	B
	Dicamba	2.065 (0.755)	10.027 (1.523)	A
	Untreated	0.206 (0.132)		
		Herbicide	0.0034	
		TOA	0.37	
		Herbicide*TOA	0.34	

Table 1. Expression of *ACS1* resulting from 2,4-D and dicamba applications relative to untreated control with experiments presented separately, 2018.

[a]NRQ = normalized relative quantity of transcript. Normalized by dividing relative quantity of *ACS1* transcript by relative quantity of *18SRibo*, the reference gene.

[b]SE = standard error of the mean. Standard error for relative expression means calculated as described by the equation:

$$SE\left(\frac{NRQ}{NRQunt}\right) = \left[\frac{NRQ\text{^}2}{NRQunt\text{^}2}\left(\frac{SE(NRQ)\text{^}2}{NRQ\text{^}2} + \frac{SE(NRQunt)\text{^}2}{NRQunt\text{^}2}\right)\right]^{1/2}$$

where *SE* is the standard error of corresponding terms, *NRQ* is the mean normalized relative quantity for each respective treatment, and *NRQunt* is the mean normalized relative quantity for the untreated control.

[c]Means followed by different letters differ significantly according to student's t-test at $\alpha = 0.05$.

[d]TOA = time of application.
