# Supplementary material for: Investigation of physiological and molecular mechanisms conferring diurnal variation in auxinic herbicide efficacy
Source: PLoS One. 2020 Aug 28;15(8):e0238144. doi: 10.1371/journal.pone.0238144 (PMC7454982; doi:10.1371/journal.pone.0238144)
Supplement: S1 File — (PDF) [file pone.0238144.s011.pdf]

<u>Supporting Information</u>

Models Tested for Translocation Data

The exponential decay function is given by the formula:

$$y = T_{min} + (T_0 - T_{min})(exp(-x/e)) \quad [1]$$

where y is translocation out of the treated leaf, T_{min} is the lower limit, or minimum, of translocation, T_0 is y at $x = 0$, and e is the steepness of decay. The log-logistic function is given by the formula:

$$y = T_{min} + [(T_0 - T_{min})/(1 + exp(b(log(x) - log(I_{50}))))] \quad [2]$$

where y is translocation out of the treated leaf, T_{min} is the lower limit, or minimum, of translocation, T_0 is y at $x = 0$, b is the slope, and I_{50} is the inflection point or dose giving 50% response. The asymptotic regression function is given by the formula:

$$y = T_0 + (T_{max} - T_0)(1 - exp(-x/e)) \quad [3]$$

where y is translocation out of the treated leaf, T_0 is y at $x = 0$, T_{max} is upper limit of translocation, and e is the steepness of increase.

CTAB Buffer Recipe

**Reagent**	**Concentration**
Cetyltrimethyl ammonium bromide (CTAB) (10% in H_2O)[a]	3%
5 M NaCl	28%
0.5 M EDTA (pH 8.0)	4%
1 M Tris-Cl (pH 8.0)	10%
Polyvinylpyrrolidone (PVP) (MW 40 kDa)	3%
β-Mercaptoethanol	0.2%
H_2O	24.8%

Initial primers designed from multiple scaffold alignment of the *A. thaliana NCED3* gene and *A. hypochondriacus* genome

Sequence Name	Sequence	GC	T_m
		%	°C
NCED3F1	GGTCATCATTTCTTTGACGGTGA	44	55
NCED3R1	AATCCAGACACCTTTGGCCA	50	57
NCED3F2	TGAAACCCACCGGTTTAAACAAGAA	40	57
NCED3R2	TAAGGAACTCTTGACGGAAGCTT	44	56

Multiple *NCED3* sequence alignment for scaffolds 71, 28, and 373 in *Amaranthus hypochondriacus*, made using Clustal Omega 0(1.2.4)

```
AHYPO_017265-RA      ------------------------------------------------------------      0
AHYPO_008268-RA      ----------ATGA-----------TCATCATGAATCCTACAATAACTCCTACATCAACC      39
AHYPO_004405-RA      ATGGCATCAATTAGTAATACTAATTGGGTAATTAAACCCCAAATTACCTCTAAATCAAAG      60


AHYPO_017265-RA      ------------------------------------------------------------      0
AHYPO_008268-RA      A------------ATACAACTACAATAAATATAC--------CCTACAAAGCCCATTTAC      79
AHYPO_004405-RA      CTTCATTTGGGTTCTAATCCTACCCTTAAGTGGCCTAAAAAAGCTACCCATTCTATTAAC      120


AHYPO_017265-RA      ------------------------------------------------------------      0
AHYPO_008268-RA      CCATCTCTTCTAAGCCCAT-----TTTCAGCCCGAAAAAGCCCATTACCATTCATTGTGC      134
AHYPO_004405-RA      TCTGTTCTTCAATTACCTTTACTTGATTCTCCTAAAAAATCCAATATCCAACCC------      174


AHYPO_017265-RA      ------------------------------------------------------------      0
AHYPO_008268-RA      TCTTCACTCAGCAGCCCCTCCTTCCATTTTCCATTT---CCCAAACCATGAATCATCACC      191
AHYPO_004405-RA      -------AAATCCCCACCTACTAAAAATTTCCCTACAAATCCTTCTACTCTTAATCCCCC      227


AHYPO_017265-RA      ------------------------------------------------------------      0
AHYPO_008268-RA      CAAATCTTCTCCTCCTCATCATCTTCCTCAATGGAACCTTTTCCAAAAAGCTGCTGCATC      251
AHYPO_004405-RA      CAAAAAATCATCGAATCCTCTACCTCATCAATGGAACCCAATTCAAAAAGCTGCTTCAAT      287


AHYPO_017265-RA      ------------------------------------------------------------      0
AHYPO_008268-RA      AACCTTTGACTTCATTGAAAACACCCTTACAACCCGAGAACGTGCCCACCCGTTTCCCAA      311
AHYPO_004405-RA      TGCTTTAGACATGGTGGAAAATGCCATAAATTCCTTTGAAAACCAACACCCACTTCCAAA      347


AHYPO_017265-RA      ------------------------------------------------------------      0
AHYPO_008268-RA      AACATCCGACCCGAAAATCCAAATATCCGGTAACTTCGCCCCAGTACAAGAAACCCCCGT      371
AHYPO_004405-RA      AACATCCGACCCGAGGGTCCAAATATCCGGTAATTTCGCCCCGGTACCTGAACAACCCGT      407


AHYPO_017265-RA      ------------------------------------------------------------      0
AHYPO_008268-RA      CAAACAATCTCTCCCTATCATAGGAGAAATACCATCTTGCATTGACGGCGTTTACGTACG      431
AHYPO_004405-RA      TAAAAAGAACCTTCCAGTTATCGGGTCTATCCCAGAATGTATCCGTGGAGTGTACGTTAG      467


AHYPO_017265-RA      ----------------ATGCTACCACCCTCTGGTGCCCATCACTTGTTTGACGGTGATGG      44
AHYPO_008268-RA      AAACGGCGCTAACCCTTTATTCAAACCAACCGCCGGCCACCATTTATTTGACGGTGACGG      491
AHYPO_004405-RA      AAACGGAGCTAACCCACTTTACGAACCCGTAGCCGGTCATCATTTCTTTGACGGTGACGG      527
```

```
                                       *      ***    *  *  ** ** ** *********** **

AHYPO_017265-RA       GATGATCCACGCCGTTAAACTTGGACCGGGAAATAAAGCCTCTTACTGCTGCCGGTTTAC       104
AHYPO_008268-RA       CATGGTTCACGCCGTTACCATCCACAACGG---GGTAGCAAGTTACGCCTGTAGATTCAC       548
AHYPO_004405-RA       AATGATTCACGCCGTTCAGTTTAACTCCGATGGGTCTGTGAATTACTGTTGCCGGTTCAC       587
                       *** * *********    *       *        *    ****   **  * ** **

AHYPO_017265-RA       TAAAACAAACCGGTTTGTTCAAGAAAAAGCAGCCGGGAGACAGTTATTCCCTAAACCGGT       164
AHYPO_008268-RA       TGAAACCAACAGATTAACGCAAGAACGGGATCTGGGTCGAGCTGTTTTCCCTAAATCTAT       608
AHYPO_004405-RA       TGAAACCCACCGGTTTAAACAAGAACGGGAGTTGGGTCGACCCATTTTCCCTAAAGCAAT       647
                      * ****  ** * **    ******   *     **  **    * ********* *  *

AHYPO_017265-RA       TAGTGAATTGCACGGTCAAACCGGATTGCTTAGGCTGGGTTTATTTTATGCACGTGTCGC       224
AHYPO_008268-RA       CGGTGAACTCCATGGTCATTCAGGAATTGCTAGATTGTTTTTGTTTTACGCTCGTGGGTT       668
AHYPO_004405-RA       CGGTGAATTACATGGGCATTCGGGTATCGCCCGTCTTCTTTTGTTCTACTCCCGTGCTCT       707
                        ***** * ** ** **  * **  *     *  *   *** ** **  * ****

AHYPO_017265-RA       TGTTGGGTTAATTAATCCGTCACGTGGGACAGGTGTCGCTAATGCTGGGTTAGTTTATTT       284
AHYPO_008268-RA       GTTTGGGCTTTTGAATCATGAAAATGGTATGGGTGTTGCTAATGCTGGTTTAGTTTATTT       728
AHYPO_004405-RA       GTTCGGTCTACTAGATCAAAACAATGGTATAGGAGTAGCAAACGCTGGAGTAGTTTATTT       767
                        * **  *  *  ***       *** *  ** ** ** ** *****  **********

AHYPO_017265-RA       TAACGGAAGATTGCTTGCTATGTCCGAGGACGACTTGCCATATCATGTTAAAATCAACGG       344
AHYPO_008268-RA       TAATGGAAGGTTGTTAGCTATGTCAGAAGATGATATACCTTACCATGTAAAAGTTACCTC       788
AHYPO_004405-RA       CAATAACAGATTATTAGCTATGTCGGAAGATGATTTACCTTATCAAATTCAAATTACTCC       827
                       **    ** **  * ******** ** ** **  * ** ** **  *  ** * *

AHYPO_017265-RA       CGATGGTGATTTGATTACGATCGGACGGTTTAATTTTAACGAGCAGGTAAACTGCCCTTT       404
AHYPO_008268-RA       TAATGGCGATTTAAAAACTGTGGGAAGGTTTGATTTCAATGGGCAGTTAAATTCCTCCAT       848
AHYPO_004405-RA       TTCAGGTGATCTGAAAACTGTTGGTAGATATGGATTTAATGGCGAATTGAAATCAACAAT       887
                          ** *** * *  **  * **  * * *   ** ** *   *  * ** *   *  *

AHYPO_017265-RA       GATAGCACACCCTAAGGTGGACCCTATCACAGGGGATTTTCATACGTTAAGCTACGACGT       464
AHYPO_008268-RA       GATTGCTCACCCAAAACTTGATCCACAATCCAAAGAATTATTTGCTTTAAGTTACGATGT       908
AHYPO_004405-RA       GATTGCGCATCCAAAAATCGATCCGGTTAGTCATGAAATGTTCGCTTTAAGCTACGATGT       947
                      *** ** ** ** **  * ** **          **  *     * ***** ***** **

AHYPO_017265-RA       CTTACGTAGACCGTATCTTAAATACTTCAGGTTCGATAAGAAGGGCCAGAAATCTCGTGA       524
AHYPO_008268-RA       TATTAAACCTCCATTTCTTAAATACTTTAAATTTTCACGGGATGGTGAGAAATCTGAAGA       968
AHYPO_004405-RA       TGTGAAGAAACCCTATTTGAAATACTTCTATTTCAAAGAAAATGGTACGAAATCTGCTGA       1007
                        *       ** * * * ********    **        * **   *******   **

AHYPO_017265-RA       GGTACACGTGCCTCTTACAAAGCCTACTATGATTCATGACTTTGCTATAACTAAAAACTA       584
AHYPO_008268-RA       TGTTGAAATCCCATTAAAGGAACCTACAATGATGCATGATTTCGCTATTACCCAGAATTT       1028
AHYPO_004405-RA       TGTTGAAATTGATCTTAAATCACCAACTATGATGCATGATTTCGCAATTTCGGAGAATTT       1067
                       **  *  *     * *     ** ** ***** ***** ** ** **  *  * ** *

AHYPO_017265-RA       CGTAATCATCCCGGATAATCAAGTTGTATTCAAGCTATCCGAAATGGTTTGGGGCGGGTC       644
AHYPO_008268-RA       TGTAGTTATCCCAGATCAACAGGTGGTGTTTAAGATGAAGGAAATGATTACCGGAGGATC       1088
AHYPO_004405-RA       CGTTATAATTCCTGATTCACAAGTTGTGTTTAAGCTTCAAGAAATGATCCATGGCGGTTC       1127
                       **  * ** ** ***   ** ** ** ** *** *    ****** *    ** ** **

AHYPO_017265-RA       ACCTCTCGGGTTTGACCCAAATAAAAAGGCCCGATTTGGGATTCTTTCAAAGGAGGATAC       704
AHYPO_008268-RA       TCCCGTTGTTTTTGACAAGAACAAAACGTCGCGTTTTGGGGTTATGAGAAAAGACGCTAA       1148
AHYPO_004405-RA       ACCTGTTGTTTTCGATAAAGAGAAGGTTTCTCGATTCGGAATTCTCCCTAAATATTCAAA       1187
                       **  * *  ** **     * **     * ** ** **  ** *    **  *    *

AHYPO_017265-RA       AAATGGGTTGAAGATAAAATGGGTTGAAGTTCCGAATAGCTTTTGCTTTCATTTTATAAA       764
AHYPO_008268-RA       AAAAGCGTCGGAAATTGTATGGGTTGATTCTCCTGACACTTTTTGCTTCCATGTTTGGAA       1208
AHYPO_004405-RA       ATCTTCCGATGAAATTCAATGGATTGATGTTCCAGATTGTTTTTGCTTTCATTTATGGAA       1247
                      *          * **   **** ****   ***  *    ******** *** *    **
```

```
AHYPO_017265-RA      TGCATGGGAAGAAAATGAAGGTAGAAATATAGTTATTATCGGGTCATGCATGAGCCCACC      824
AHYPO_008268-RA      CGCTTGGGAAGAATCTCAAAGCGACGAGATTGTGGTTATTGGGTCTTGTATGACTCCTCC      1268
AHYPO_004405-RA      TGCTTGGGAAGAATCTGACTCCGATGAGATCGTCGTAATTGGATCTTGTATGACTCCGGC      1307
                      ** *********  * *        * ** **  * ** ** ** ** ****  **  *

AHYPO_017265-RA      CGATTCAATATTTACCGAGAGTGATGAACCGGCCCGAATAGAGCTTTGTGAAATCGGGTT      884
AHYPO_008268-RA      TGATTCTATTTTTAATGAATGTGATGAAAATTTAAAGAGTGTTTTATCTGAAATCCGGCT      1328
AHYPO_004405-RA      CGACTCCATTTTCAACGAATGTGACGAGAATCTCTCTAGTGTGTTGTCCGAAATCCGATT      1367
                      ** ** ** ** *  **  **** **          *  *   * *  ****** *  *

AHYPO_017265-RA      GGATGTCGAAACGGGTAAATCAACCCGACGGGTGATAGTTTT---------------GGG      929
AHYPO_008268-RA      TAATTTAAGGACGGGTCAGTCAACCCGACGACCCATTATTGACCCGGCCCTGTATGATCA      1388
AHYPO_004405-RA      AAACCGTAAAACCGGAGTTTCAACCCGGAGAGAAATCCTTCCG------AATTCGGAGAA      1421
                       *       ** **    ********  *    **  **

AHYPO_017265-RA      TATGAATTTAGAAGTAGGGCAAGTAAATAAAAAGCTCTTAGGAAGAAAGACAAGGTACGT      989
AHYPO_008268-RA      TTTTAATCTTGAAGCGGGTATGGTAAACCGTAATTTACTTGGGAGAAAAACCCGGTTTGC      1448
AHYPO_004405-RA      AATGAATTTAGAAGCGGGAATGGTGAATAAGAACAGACTTGGAAGAAAAACACAATTTGC      1481
                       * *** * ****  **    ** **    **     * ** ***** **    *  *

AHYPO_017265-RA      TTATATGGCAATAGCCGATTCATGGCCAAAATGTGGTGGTATTGCTAAGGTTGATTTAGT      1049
AHYPO_008268-RA      TTATTTAGCAATTGCTGAACCGTGGCCTAAAGTTTCGGGGTTTGCTAAGGTTGATTTGGA      1508
AHYPO_004405-RA      TTATCTCGCCATTGCTGAACCATGGCCAAAGGTGTCTGGATTCGCTAAAGTAGATTTAAT      1541
                     **** * ** ** ** **  * ***** **       **   * ***** ** *****

AHYPO_017265-RA      GAATGGGAAAGTGAATAAGTATATGTATGGGCATGATAGATATGGTGGGGAACCTTGCTT      1109
AHYPO_008268-RA      AACGGGTGAGGTAAAAAAATTTATTTACGGTGGTGAAAAGTACGGTGGTGAACCTTTCTT      1568
AHYPO_004405-RA      CAATGGAGAAGCAAAGACATTCTTGTATGGAGAACACAAATATGGCGGAGAACCATTATT      1601
                      *  **  * *  ** *  *   * ** **     * *  ** ** ** ***** *  **

AHYPO_017265-RA      TGTTCCGGCGAATGCAAATAATAATAATAATAATAATAATAATAATAATAATAATGAATT      1169
AHYPO_008268-RA      TTTACCGCGAGGTAATAATAGTTTTGATAGTGATTCTGAGGATGATGG------------      1616
AHYPO_004405-RA      TCTTCCTAAAAATGG---------------CGAAACAGAAGATGATGG------------      1634
                     * * **      *                   *     *  ** **

AHYPO_017265-RA      AGGAAATGAAGATGAAGGATGGATAATTAGTTTAGTGAGGGATGAAAATGTAGAGAGATC      1229
AHYPO_008268-RA      ------------------GTATGTTTTGGGATTTGTACATGATGAAAAGGAAGGGAGTTC      1658
AHYPO_004405-RA      ------------------TTACATTCTTGCATTTGTTCATGATGAGAAGAATCAAGAATC      1676
                                        *   *  *    ** **    ***** **          **

AHYPO_017265-RA      AGAGTTGGTGATATTAAGAGCTAAGGATATGAAACAAATAGGATGTGTATGTATGCCTTC      1289
AHYPO_008268-RA      GGAATTGTTAATTGTAAATGCAAGTAATTTGGAAGTTGAAGCTTCGATTAAGTTGCCTTC      1718
AHYPO_004405-RA      AGAGCTTCAAATAGTCAACGCCATGGATTTAGAATTGGTTGCGACTGTCAAGCTTCCGTC      1736
                      **  *    **  * *  ** *   ** *  **      *      *     * ** **

AHYPO_017265-RA      TAGGGTTCCTTATGGGTTTCATGGCACATTTCTTGATCAAAACCAATTAAGTGTACAAAC      1349
AHYPO_008268-RA      TAGAGTTCCTTACGGTTTTCATGGAACGTTCGTGCGATCAGAAGATTTGCAAGATCAAGA      1778
AHYPO_004405-RA      AAGAGTTCCTTACGCGGGTTAA--------------------------------------      1758
                      ** ******** *    * *

AHYPO_017265-RA      AAATCATTAA---------    1359
AHYPO_008268-RA      TAAAGTTTGCGCCGTTTAA    1797
AHYPO_004405-RA      -------------------    1758
```

Reverse strand of coding sequence for *NCED1* product obtained from Sanger capillary sequencing (A) and associated protein sequence at 3rd reading frame (B).

A)
GTGACGGAATGATTCACGCCGTTCAGTTTAANTNGGATGGGTCTGTGAATTACTGTTGCCGG
TTCACGGAAACCCACCGGTTTAAACAAGAACGGGAGTTGGGCCGACCCATTTTCCCTAAAGC
AATTGGTGAATTACATGGGCATTCGGGTATTGCCCGTCTACTATTGTTCTACTCCCGTGCTCT
GTTCGGTCTGCTAGATCAAAACAATGGTATAGGAGTAGCAAACGCTGGAGTAGTTTATTTCA
ATAACAGATTATTAGCTATGTCGGAAGATGATTTACCTTATCAAATTCAAATTACTCCGTCA
GGTGATCTGAAAACTGTTGGTAGATATGGATTTAATGGAGAATTGAAATCGACAATGATTGC
GCATCCGAAAATCGATCCGGTTAGTCATGAAATGTTCGCTTTAAGCTACGATGTTGTGAAGA
AACCCTATCTGAAATACTTTTATTTCAAAGAAAATGGTACGAAATCTGCTGATGTTGAAATT
GATCTTAAATCACCAACTATGATGCATGATTTCGCAATTTCGGAGAATTTCGTTATAATTCCT
GATTCACAAGTTGTGTTTAAGCTTCAAGAAATGATCCATGGCGGTTCCCCTGTAGTTTTCGAT
AAAGAGAAGGTTTCGCGATTCGGAATTCTCCCTAAATATTCAAAATCTTCCGATGAAATTCA
ATGGATTGATGTTCCAGATTGTTTTTGCTTTCATTTGTGGAATGCTTGGGAAGAATCCGACTC
CGATGAGATCGTCGTAATCGGATCTTGTATGACTCCGGCCGACTCCATTTTCAACGAATGTG
ACGAGAATCTCTCTAGTGTGTTGTCCGAAATCCGATTAAACCGTAAAACCGGAGTTTCAACC
CGGAGAGAAATCCTTCCGAATTCGGAGAAAATGAATTTAGAAGCAGGAATGGTGAACAAGA
ACAAACTCGGAAGAAAAACACAATC

B)
DGMIHAVQFXXDGSVNYCCRFTETHRFKQERELGRPIFPKAIGELHGHSGIARLLLFYSR
ALFGLLDQNNGIGVANAGVVYFNNRLLAMSEDDLPYQIQITPSGDLKTVGRYGFNGELKSTMIA
HPKIDPVSHEMFALSYDVVKKPYLKYFYFKENGTKSADVEIDLKSPTMMHDFAISENFVIIPDSQ
VVFKLQEMIHGGSPVVFDKEKVSRFGILPKYSKSSDEIQWIDVPDCFCFHLWNAWEESDSDEIVVI
GSCMTPADSIFNECDENLSSVLSEIRLNRKTGVSTRREILPNSEK
MNLEAGMVNKNKLGRKTQ

(Sequencing primers used in reaction mixture: F 5’-GGTCATCATTTCTTTGACGGTGA-3’ and R 5’-AATCCAGACACCTTTGGCCA-3’)

Specific qPCR primers tested and used for quantification of transcript levels, 2018.

Sequence Name	Sequence	GC	T_m[†]	Amplicon Length
		%	°C	bp
NCED1F1	GGTATTGCCCGTCTACTATTG	48	60	138
NCED1R1	AGGTAAATCATCTTCCGACATAG	39	60	--
NCED1F2	GATCGTCGTAATCGGATCTTG	48	60	126
NCED1R2	TCTCTCCGGGTTGAAACT	50	60	--
NCED1F3	TTACTGTTGCCGGTTCAC	50	60	135
NCED1R3	GCACGGGAGTAGAACAATAG	50	60	--
ACS1F1	GGAGAAAGTGAGAGGCAATAAG	46	61	144
ACS1R1	GCAGGATAGTAAGGTGTAGGA	48	60	--
ACS1F2	GCTTCGATCTGGTGAAAGAG	50	60	119
ACS1R2	GTGTGGTAAACCGTGGTAAT	45	60	--
ACS1F3	AAGCTGGATGGTTTAGAGTATG	41	60	138
ACS1R3	GATGCCAACATTTCCTCTTTG	43	60	--
18SRiboF1	CCTTACGGACGAGCTATTG	53	59	132

18SRiboR1	GAGCACGCTCAAGTTCAT	50	60	--
18SRiboF2	AGTGGATGCACCCAGTATT	47	61	128
18SRiboR2	TCGATGGTTCACGGGATT	50	61	--

Melting temperatures are based on SYBR Green master mix (intercalating dye) used for qPCR.
