# Supplementary material for: Investigation of physiological and molecular mechanisms conferring diurnal variation in auxinic herbicide efficacy
Source: PLoS One. 2020 Aug 28;15(8):e0238144. doi: 10.1371/journal.pone.0238144 (PMC7454982; doi:10.1371/journal.pone.0238144)
Supplement: S1 Raw images — (PDF) [file pone.0238144.s012.pdf]

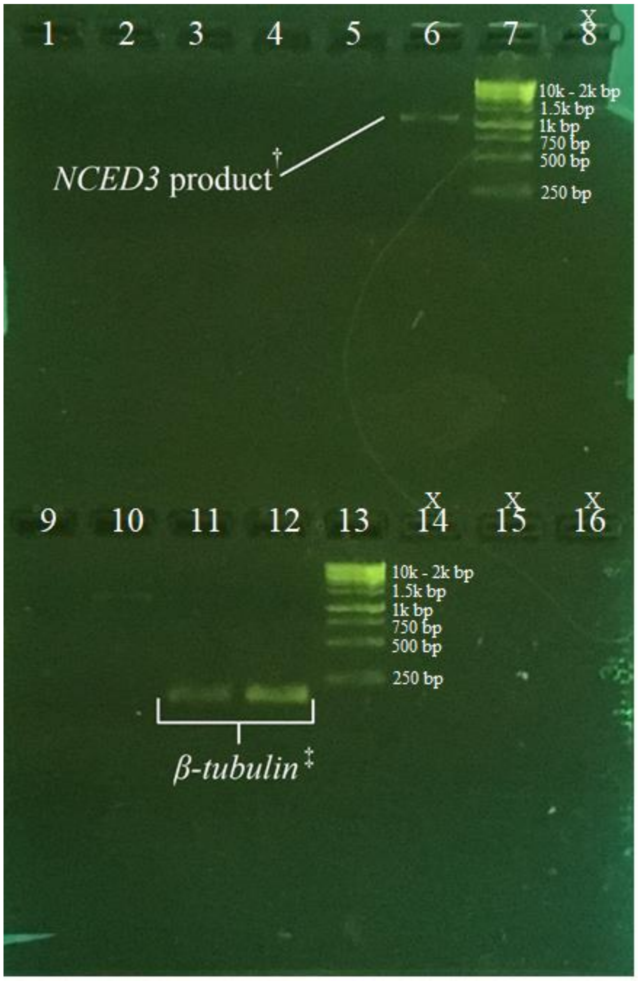

Loading Order, primers designed from scaffold alignments:

Lanes 1 and 2 - *ACS1* initial test primers*
F - AGCAGCTAACAATGGACATGG
R - CAATGACATGATGATCCTGGTGAAAC

Lanes 3 and 4 - *ACS1* initial test primers*
F - ACTTCCTTGGTTGGGAAGAATATGA
R - GTTTTGCTAGGAGGTATTGGGTTTG

Lanes 5 and 6 - *NCED3* initial test primers
F - GGTCATCATTTCTTTGACGGTGA
R - AATCCAGACACCTTTGGCCA

Lane 7 - ladder (1 µl)

Lanes 9 and 10 - *NCED3* initial test primers
F - TGAAACCCACCGGTTTAAACAAGAA
R - TAAGGAACTCTTGACGGAAGCTT

Lanes 11 and 12 - *β-tubulin* reference test primer
F - ATGTGGGATGCCAAGAACATGATGTG
R - TCCACTCCACAAAGTAGGAAGAGTTCT

Lane 13 - ladder (1 µl)

All test primers were run with 10 µl of mixture used in PCR, with primer concentrations making up 0.2 µM concentration. Contents in addition to diluted primers: 2 µl of cDNA extract from 2,4-D treated plants, 2 µl 5X DNA polymerase buffer, 0.2 µl of 10 mM dNTPs, 0.6 µl of 25 mM $MgCl_2$, 0.05 µl of Taq polymerase, 3.15 µl of autoclaved distilled water

Experimental samples used for primer testing consisted of cDNA originating from RNA extracted from leaf tissue in *A. palmeri* (Lanes 1-6, 8-11)

Image captured using an iPhone 6S with no filters or adjustments

Corresponds to Figure S5

*Test primers for *ACS1* were run in this gel because at the time of primer testing, sequence for *ACS1* in *A. palmeri* had not been obtained yet from Giacomini et al. (2019). Therefore, *ACS1* primers shown here were not used for experiment contained in manuscript.

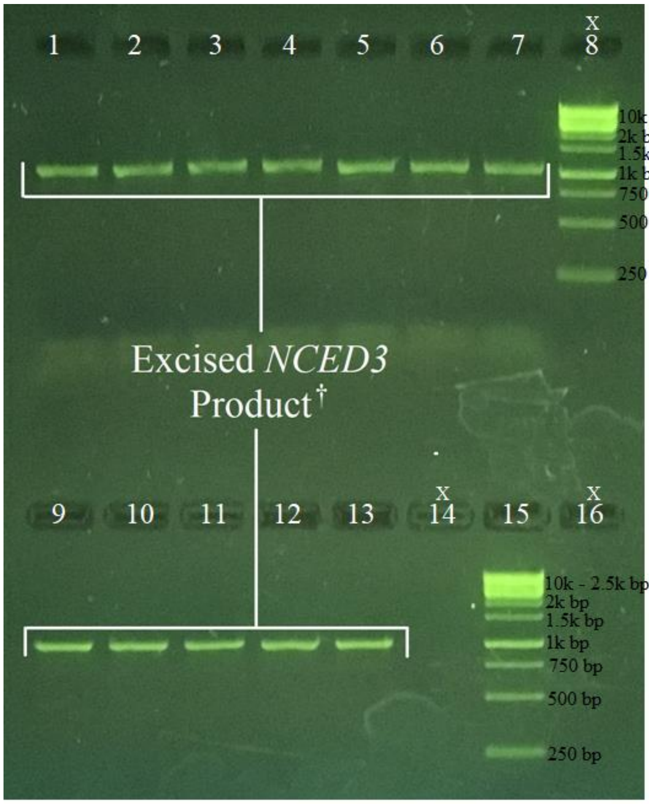

Loading order, *NCED3* gel excision:

Lanes 1-7, 9-13: *NCED3* test primer pair 1
F - GGTCATCATTTCTTTGACGGTGA
R - AATCCAGACACCTTTGGCCA

Lanes 8, 15: ladder (1 µl)

All test primer pairs were run with 20 µl of mixture used in PCR, with primer concentrations making up 0.4 µM concentration. Contents in addition to diluted primers: 4 µl of cDNA extract from 2,4-D treated plants, 4 µl 5X DNA polymerase buffer, 0.4 µl of 10 mM dNTPs, 1.2 µl of 25 mM $MgCl_2$, 0.10 µl of Taq polymerase, 6.3 µl of autoclaved distilled water

Experimental samples used for primer testing consisted of cDNA originating from RNA extracted from leaf tissue in *A. palmeri* (Lanes 1-7, 9-13)

Image captured using an iPhone 6S with no filters or adjustments

Corresponds to Figure S6
