# Supplementary material for: Investigation of physiological and molecular mechanisms conferring diurnal variation in auxinic herbicide efficacy
Source: PLoS One. 2020 Aug 28;15(8):e0238144. doi: 10.1371/journal.pone.0238144 (PMC7454982; doi:10.1371/journal.pone.0238144)
Supplement: S1 Data — (ZIP) [file pone.0238144.s013.zip › Data/Ethylene Experiments/Final Analyses.docx]

| 2,4-D |  | | | | |
| --- | --- | --- | --- | --- | --- |
| Treatment | Mean (SE) | | Equation | Slope (SE) |  |
|  | µL kg FW^-1^ h^-1^ | |  |  |  |
| 1:00 PM | 2.36 (0.15) | CD | y = 1.86 + 0.019x | 0.0194 (0.0044) |  |
|  |  |  |  |  |  |
| 8:00 AM | 2.37 (0.14) | BCD | y = 1.96 + 0.019x | __ |  |
|  |  |  |  |  |  |
| 8 am + TIBA | 2.69 (0.14) | A | y = 2.18 + 0.019x | __ |  |
|  |  |  |  |  |  |
| 8 am + NPA | 2.35 (0.14) | D | y = 1.84 + 0.019x | __ |  |
|  |  |  |  |  |  |
| 8 am + Verapamil | 2.56 (0.14) | ABC | y = 2.06 + 0.019x | __ |  |
| HAT | <.0001 |  |  |  |  |
| Treatment | 0.0019 |  |  |  |  |
| HAT*Treatment | 0.3806 |  |  |  |  |

| Dicamba |  | | | | |
| --- | --- | --- | --- | --- | --- |
| Treatment | Mean (SE) | | Equation | Slope (SE) |  |
|  | µL kg FW^-1^ h^-1^ | |  |  |  |
| 1:00 PM | 3.07 (0.07) | A | y = 2.29 + 0.037x | 0.0368 (0.0059) |  |
|  |  |  |  |  |  |
| 8:00 AM | 2.75 (0.09) | B | y = 1.97 + 0.037x |  |  |
|  |  |  |  |  |  |
| 8 am + TIBA | 2.66 (0.07) | B | y = 1.88 + 0.037x |  |  |
|  |  |  |  |  |  |
| 8 am + NPA | 3.07 (0.07) | A | y = 2.29 + 0.037x |  |  |
|  |  |  |  |  |  |
| 8 am + Verapamil | 2.87 (0.07) | AB | y = 2.10 + 0.037x |  |  |
| HAT | <.0001 |  |  |  |  |
| Treatment | 0.0004 |  |  |  |  |
| HAT*Treatment | 0.6661 |  |  |  |  |
